# Supplementary material for: Chemical design of electronic and magnetic energy scales of tetravalent praseodymium materials
Source: Nat Commun. 2023 May 30;14:3134. doi: 10.1038/s41467-023-38431-7 (PMC10229542; doi:10.1038/s41467-023-38431-7)
Supplement: Supplementary file 1 — Supplementary Information [file 41467_2023_38431_MOESM1_ESM.pdf]

Supporting Information
Chemical Design of Electronic and Magnetic Energy Scales of Tetravalent Praseodymium materials

Arun Ramanathan, Jensen Kaplan, Dumitru-Claudiu Sergentu, Jacob A. Branson
Mykhaylo Ozerov, Alexander I. Kolesnikov, Stefan G. Minasian, Jochen Autschbach,
John W. Freeland, Zhigang Jiang, Martin Mourigal, Henry S. La Pierre*

April 7, 2023

*Email: hsl@chemistry.gatech.edu

Supplementary Methods 1: Material Synthesis.

All reagents were handled in a N_2 filled glove box (Vigor) with $O_2 < 0.1$ ppm and $H_2O < 0.1$ ppm. Na_2O (Alfa Aesar), $SrCO_3$ (99.5%, Alfa Aesar), Pr_6O_{11}($\geq$ 99.5%, Alfa Aesar), Tb_4O_7 ($\geq$ 99.998%, Alfa Aesar), CeO_2 ($\geq$ 99.9%, Alfa Aesar), and Li_2O ($\geq$ 99.9%, Alfa Aesar) were used as starting materials. The metal oxides and $SrCO_3$ powders were dried by heating to 500° C for 12 h with a heating rate of 10° C/min in a box furnace (using alumina crucibles) under ambient atmosphere. The reagents were then cooled with the furnace off to $\sim$ 120° C, and then cooled to room temperature in the antechamber of the glovebox under vacuum. These dried reagents were stored in amber bottles in the glove box. An MTI-KSL-1100X-S-Ul-LD furnace was used. All crucibles were purchased from MTI.

Synthesis of 2-Ln (Ln = Ce, Pr).

Na_2LnO_3 (2-Ln, Ln = Ce, Pr) was synthesized following prior published work[1].

Synthesis of 1-Ln (Ln = Ce, Pr).

Polycrystalline powder samples of Sr_2LnO_4 (1-Ln, Ln = Ce, Pr) were synthesized using traditional solid-state methods by intimately mixing $SrCO_3$ and Pr_6O_{11} (CeO_2) in molar ratio 2.0:1 (Sr:Ln), using an agate mortar inside the glove box. The powder mixtures were pressed in to 15 mm diameter pellets outside the glovebox. The samples were fired under a flow of O_2 in tube furnace (quartz tubes with a diameter of 55 mm was used). The O_2 flow was controlled using a regulator set to 2 psig and an oil bubbler at the end of the line to $\sim$ 1 bubble every 2-3 sec. The pellets were placed on alumina boats and placed at the center of the quartz tube (lining up with the center of the heating zone in the furnace). The line was then purged with O_2 for $\sim$ 5 min. The firing was performed at 1100° C for 24 h with a cooling/heating rate of 3° C/min. O_2 flow was stopped 30 min after the furnace cooled to room temperature. The samples were taken out of the quartz tubes in air and placed into the antechamber of the glovebox as quickly as possible in order minimize contact with ambient atmosphere.

Synthesis of 0-Ln (Ln = Pr, Tb).

Polycrystalline powder samples of Li_8LnO_6 (0-Ln, Ln = Pr, Tb) were synthesized similarly to 2-Ln by intimately mixing Li_2O and Pr_6O_{11} (Tb_4O_7) in molar ratio 9.6:1 (20% excess Li_2O). Following the similar procedure to 2-Ln, the firing was performed at 700° C for 12 h with a cooling/heating rate of 3° C/min.

Supplementary Methods 2: Characterization.

Powder X-ray diffraction (PXRD).

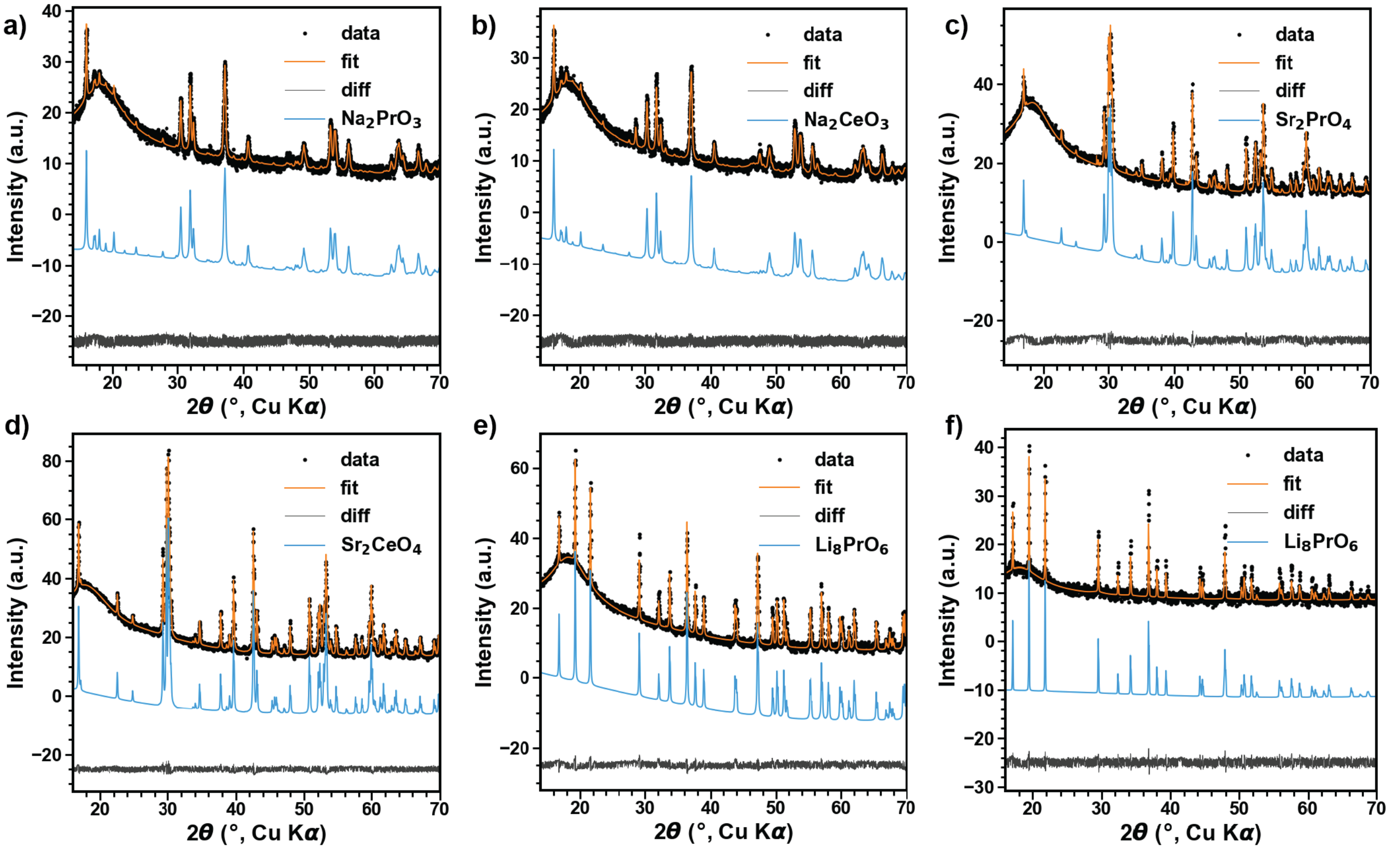

Supplementary Figure 1. PXRD of different compounds. **a**, 2-Pr (Na_2PrO_3). **b**, 2-Ce (Na_2CeO_3). **c**, 1-Pr (Sr_2PrO_4). **d**, 1-Ce (Sr_2CeO_4). **e**, 0-Pr (Li_8PrO_6). **f**, 0-Tb (Li_8TbO_6). Data is shown in black dots, Rietveld refinements in orange, the corresponding phases in blue, and the difference curves in grey. The broad hump near $2\theta = 20(^\circ)$ corresponds to polycarbonate dome background from the sample holder. All data was collected at $T = 300$ K. Quantitative Rietveld refinements to the laboratory XRD data were carried out using Bruker TOPAS 5 suite[2].

	2-Pr	2-Ce	1-Pr	1-Ce	0-Pr	0-Tb
Space group	$C_{2/c}$	$C_{2/c}$	$Pbam$	$Pbam$	$R\overline{3}$	$R\overline{3}$
Point group	D_{2d}	D_{2d}	C_{2h}	C_{2h}	S_6	S_6
a(Å)	$5.963(3)$	$6.074(3)$	$6.123(8)$	$6.118(9)$	$5.608(5)$	$5.549(6)$
b(Å)	$10.319(9)$	$10.365(6)$	$10.280(3)$	$10.349(5)$	$5.608(5)$	$5.549(6)$
c(Å)	$11.732(1)$	$11.774(8)$	$3.589(6)$	$3.597(1)$	$15.982(4)$	$15.709(7)$
$\alpha(^\circ)$	90	90	90	90	90	90
$\beta(^\circ)$	$109.9(1)$	$110.1(1)$	90	90	90	90
$\gamma(^\circ)$	90	90	90	90	120	120
d_{Ln-Ln} (Å)	$3.407(3), 3.487(6)$	D_{2d}	$3.589(6)$	$3.597(11)$	$5.608(5)$	$5.549(6)$

Supplementary Table 1. Crystal structure information.

Inelastic neutron scattering.

Broadband inelastic neutron scattering measurements using E_i = 300, 500, and 700 meV revealed a number of flat modes, common across all compounds. The flat modes are attributed to vibrational excitations and the dispersive background visible in the $E_i = 800$ meV is attributed to a hydrogen recoil line with the clear quadratic, Q^2, dependence typical of recoil processes and previously observed by Sensei $etal.$ At energies above around 400 meV, the flat modes are in the frequency range of OH stretching mode from a OH impurity in the starting materials and identified to be < 3 wt% from laboratory powder X-ray diffraction of the Na_2O and Li_2O starting materials. The OH stretches show a strong Q dependence at higher Q ruling them out as CEF transitions. After accounting for the OH stretching mode, we were able to identify clear crystal-electric field transitions in 0-Pr and 1-Pr. Broadband inelastic neutron scattering data was reduced and analyzed in MANTID on the SNS analysis cluster, ORNL. All diagonalization were carried out using pycrystalfield[3].

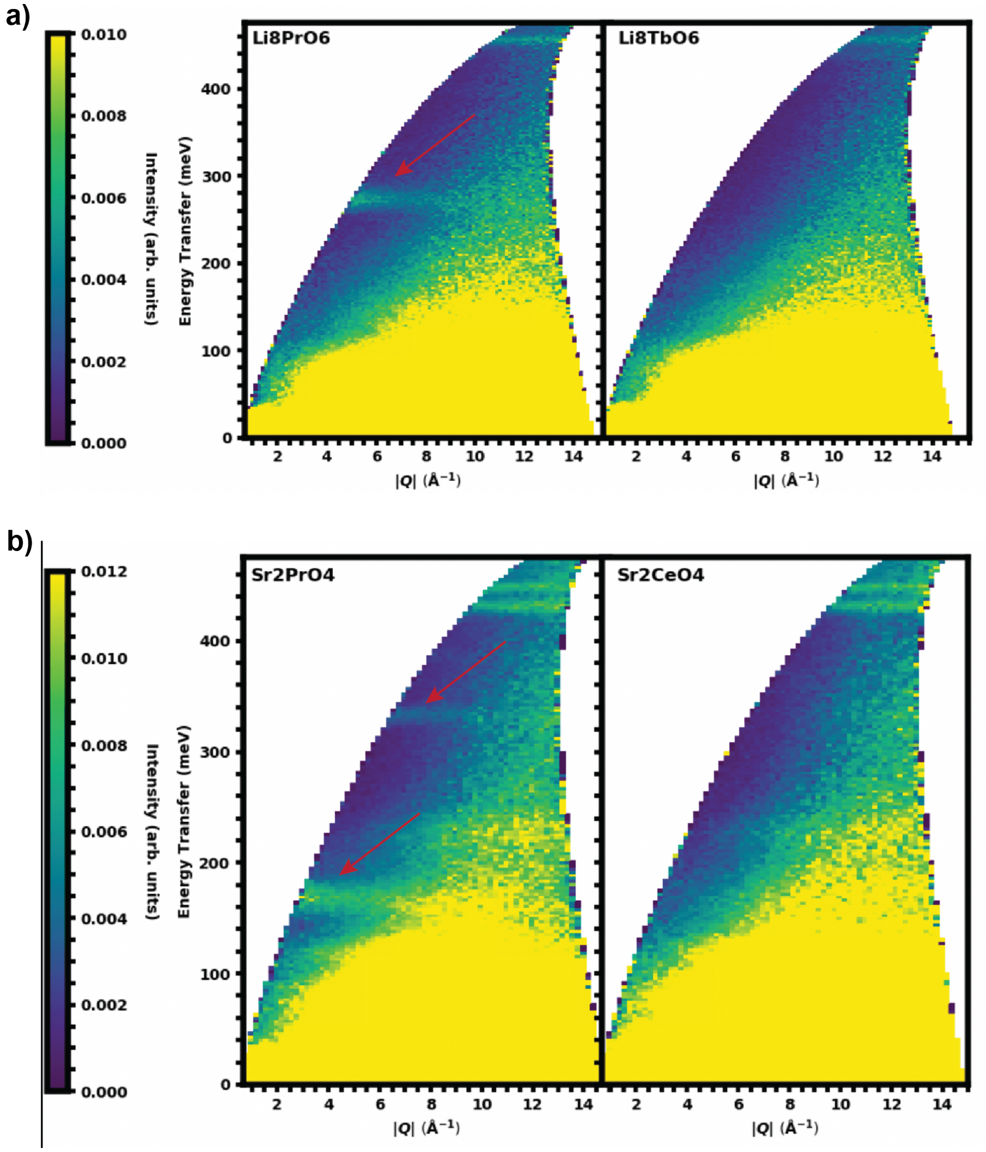

Supplementary Figure 2. Overview of INS data measured on SEQUOIA. a, 0-Ln. **b**, 1-Ln with incident energy of $E_i = 500$ meV. The CF transitions are indicated by red arrows.

STXM O K edge XAS.

The O K-edge STXM data were background subtracted using the MBACK algorithm in MATLAB. The data were normalized by fitting a first-order polynomial to the post-edge region of the spectrum and setting the edge jump at 5416 eV to an intensity of 1.0. The spectra was fit to pseudo-voigt lineshapes using in-house built python scripts. Approximate peak positions were determined using first and second derivatives of the spectrum. The edge was modeled using a step function. The TEY and TFY data were normalized to the maximum of the M_5 edge. To facilitate comparisons to previously reported O K and Pr $M_{5,4}$ edge spectra for PrO_2, the energy position of the step fucntion was optimized near the value used previously.

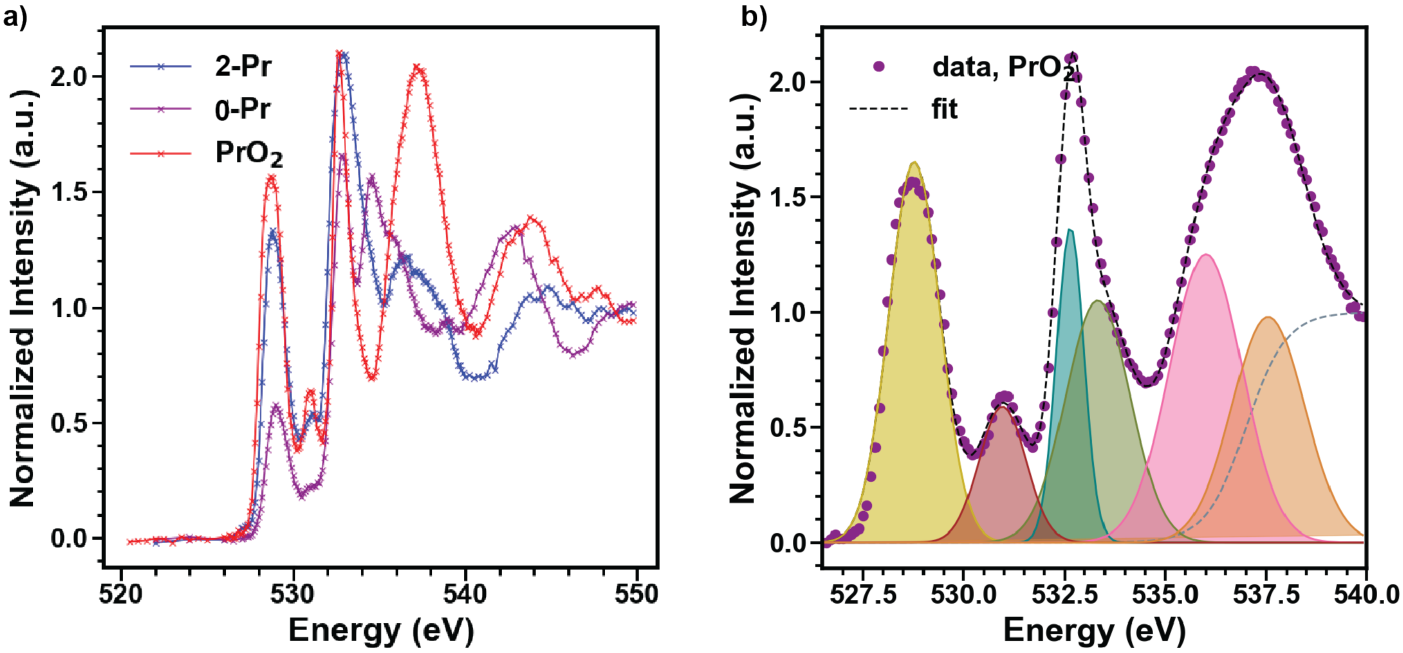

Supplementary Figure 3. STXM O K edge XAS. a, STXM O K edge data plotted together for 2-Pr, 0-Pr, and PrO_2 to show the differences in $4f$ hybridization between them. **b**, Fits to PrO_2. Data obtained from [4]. All data was collected at $T = 300$ K.

STXM Pr M edge XAS.

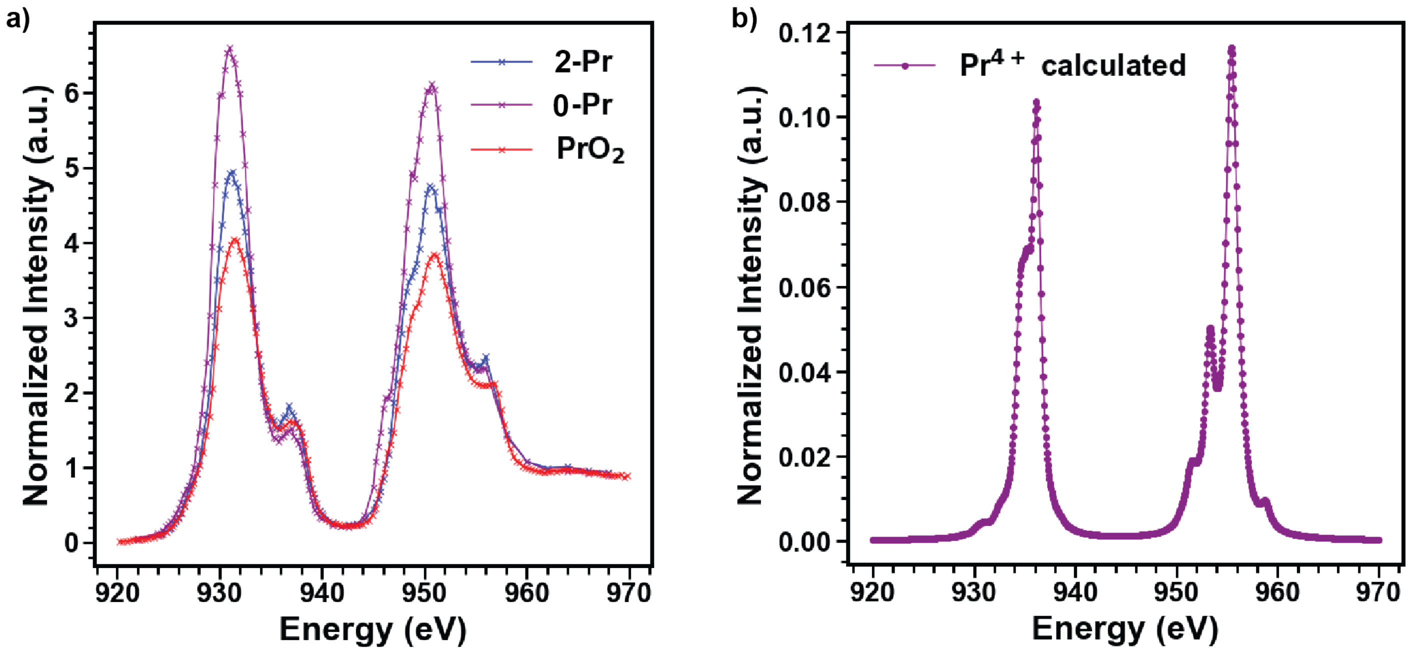

Supplementary Figure 4. STXM Pr $M_{5,4}$ edge XAS. **a**, STXM Pr $M_{5,4}$ edge data plotted together for 2-Pr, 0-Pr, and PrO_2 to show the differences in peak intensities corresponding to difference in hybridization. **b**, Pr $M_{5,4}$ edge XAS calculated using atomic multiplet theory[5] for a Pr^{4+} system which does not include Pr-$4f$/O-$2p$ hybridization. As explained in the main text, the calculation does not capture the satellite peaks and predicts a structured M_4 edge which is clearly absent in the data. PrO_2 data was obtained from[4] . All data was collected at and calculations performed at $T = 300$ K

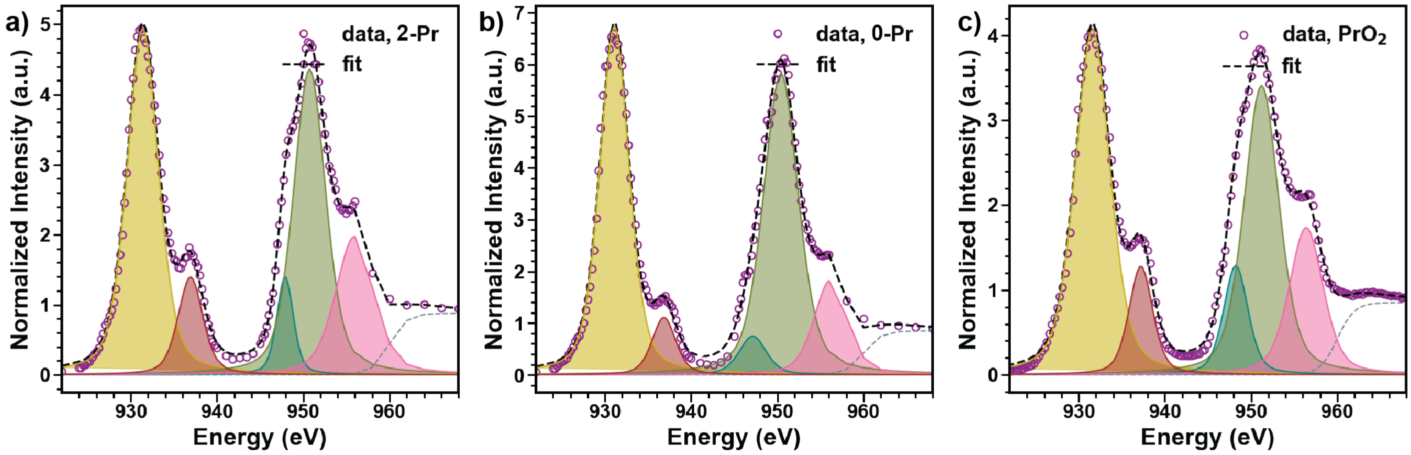

Supplementary Figure 5. STXM Pr $M_{5,4}$ edge XAS fits. **a**, 2-Pr (Na_2PrO_3). **b**, 0-Pr (Li_8PrO_6). **c**, PrO_2. Fits were carried out in in-house built python scripts using pseudo-voigt functions and setting the step jump to 1.0. The data was post-edge normalized.

	CEF ($\Gamma_7 \rightarrow \Gamma_8$, meV)^a	$I^b_{1s\rightarrow 4f}$	$\frac{I_A}{I_{A'}}^c$	$\frac{I_B}{I_{B'}}^c$
PrO_2	~ 130	$3.65(9)$	$3.176(4)$	$1.963(6)$
2-Pr	~ 168	$2.73(11)$	$3.540(4)$	$2.207(5)$
0-Pr	~ 260	$1.08(3)$	$6.062(4)$	$3.177(1)$

[a] From $abinitio$ calculations, INS, and FIRMS measurements.
[b] From O K edge XANES.
[c] From Pr $M_{5,4}$ XANES. The notations A, B and A', B' correspond to the notations in Fig3 in the main text. I also corresponds to normalized intensity of the peaks and not the integrated intensity.

Supplementary Table 2. Difference in degree of hybridization between PrO_2, 2-Pr, and 0-Pr evident from CEF transitions, O K edge, and Pr $M_{5,4}$ edge XAS.

Pr $M_{5,4}$ edge XMCD.

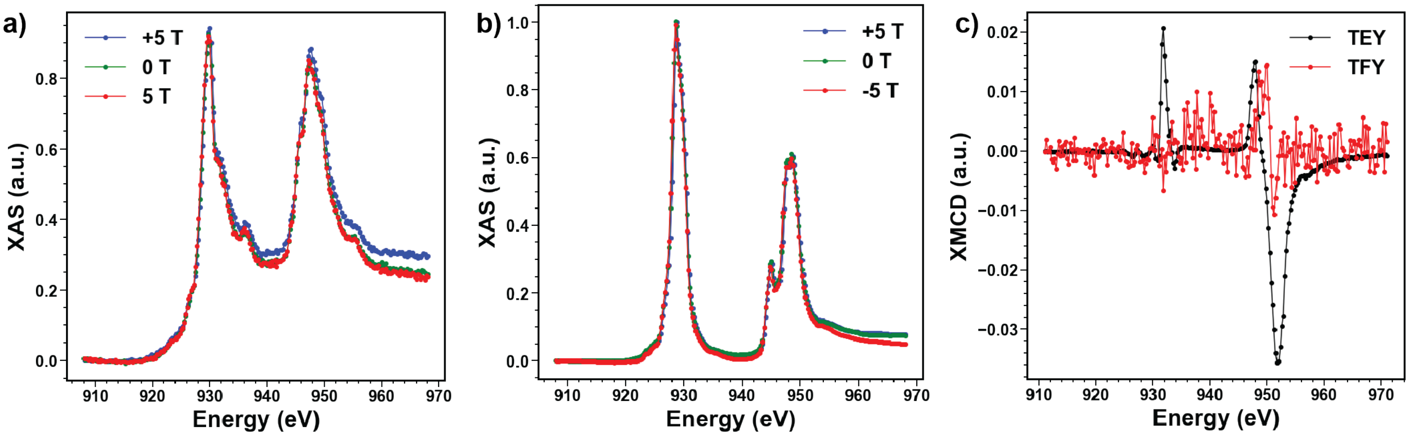

Supplementary Figure 6. Pr $M_{5,4}$ edge XMCD in 2-Pr. a, Total fluorescence yield (TFY) isotropic data measured under $\mu_o H = 0, +5, -5$ T. As expected, the TFY data looks similar to the STXM data except for a small bump at ~ 938 eV which corresponds to Cu from the sample holder. **b**, Total electron yield (TEY) isotropic data measured under $\mu_o H = 0, +5, -5$ T. The signal from Cu is not visible in the TEY data. **c**, XMCD data measured in both TEY and TFY modes. Given the poor signal of TFY, only the TEY was used for XMCD analysis in the main text. The data was normalized to the maximum of the M_5 edge.

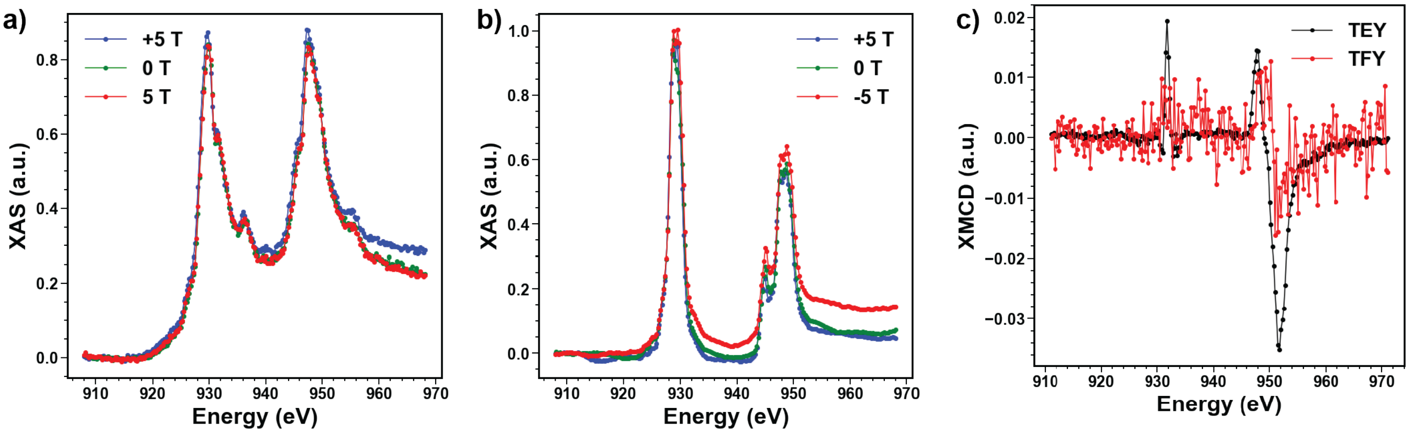

Supplementary Figure 7. Pr $M_{5,4}$ edge XMCD in 0-Pr. a, Total fluorescence yield (TFY) isotropic data measured under $\mu_o H = 0, +5, -5$ T. As expected, the TFY data looks similar to the STXM data except for a small bump at ~ 938 eV which corresponds to Cu from the sample holder. **b**, Total electron yield (TEY) isotropic data measured under $\mu_o H = 0, +5, -5$ T. The signal from Cu is not visible in the TEY data. **c**, XMCD data measured in both TEY and TFY modes. Given the poor signal of TFY, only the TEY was used for XMCD analysis in the main text. The data was normalized to the maximum of the M_5 edge.

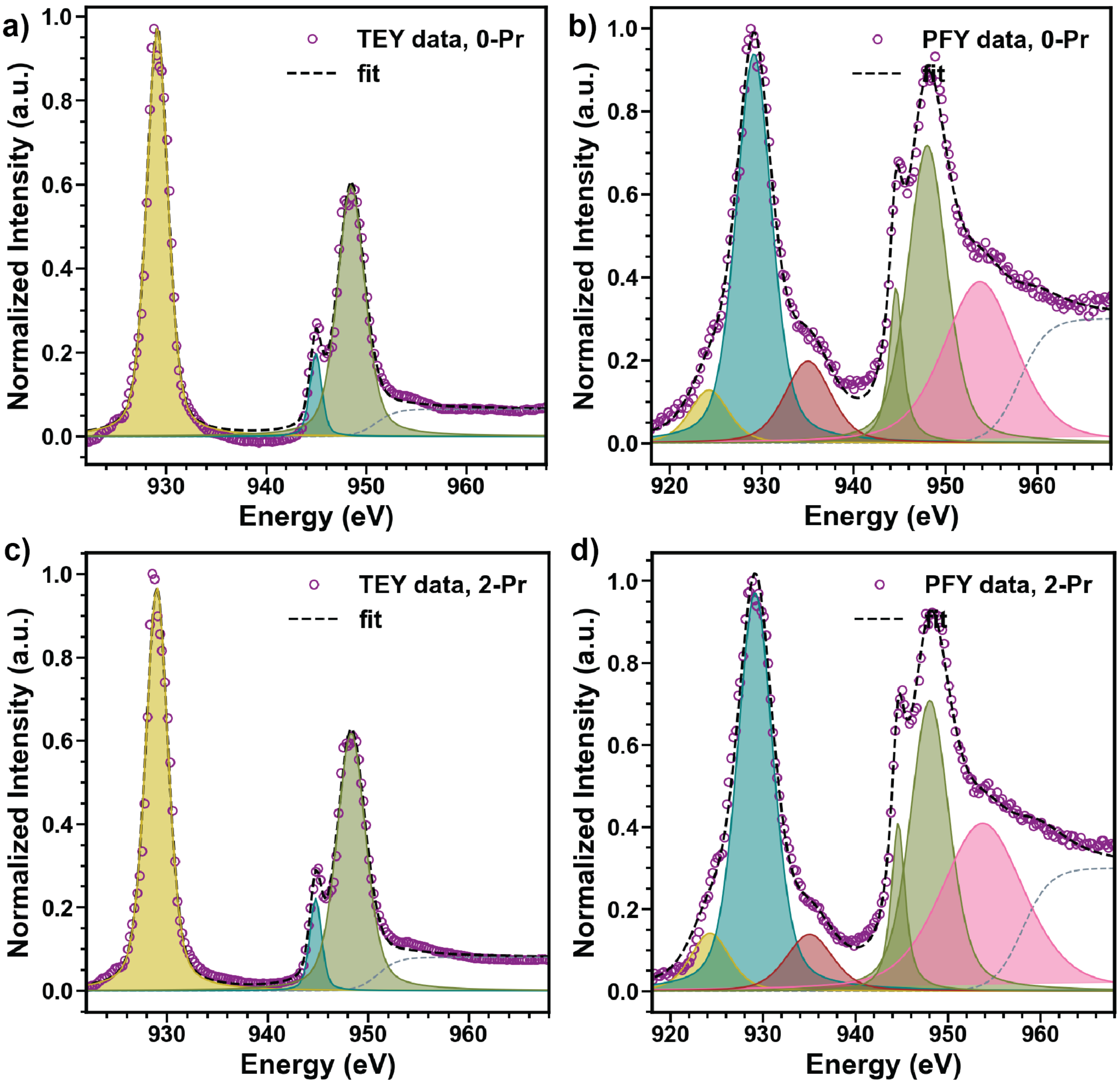

Supplementary Figure 8. Pr $M_{5,4}$ edge. a, Total electron yield (TFY) isotropic data measured under $\mu_o H = 0$ T and the corresponding fits for comparisons for 0-Pr. **b**, Partial fluorescence yield (PFY) isotropic data measured under $\mu_o H = 0$ T and the corresponding fits for 0-Pr. As expected PFY data looks similar to the STXM data. The branching ratio (BR) for TEY is 0.52(3) and for PFY is 0.43(2) in line with STXM data. **c**, Total electron yield (TFY) isotropic data measured under $\mu_o H = 0$ T and the corresponding fits for comparisons for 2-Pr. **d**, Partial fluorescence yield (PFY) isotropic data measured under $\mu_o H = 0$ T and the corresponding fits for 2-Pr. As expected PFY data looks similar to the STXM data. The branching ratio (BR) for TEY is 0.51(4) and for PFY is 0.42(3) in line with STXM data.

Supplementary Note 1: Data Analysis.

CF splitting of Pr^{4+} in $\zeta_{SOC} >> \Delta_{CF}$ regime.

The strong spin orbit coupling of a $4f^1$ ion, entangles the electron spin, $S = 1/2$ and high orbital angular momentum $L = 3$ to give rise to a $J = 5/2$ ground state ($^2F_{5/2}$) and a $J = 7/2$ excited state ($^2F_{7/2}$). The sixfold degeneracy of the $^2F_{5/2}$ ground state is removed under the crystal field. Under a highly symmetric O_h symmetry, the $^2F_{5/2}$ ground state is split into a doublet Γ_7 and a quartet Γ_8. Any deviation from the O_h symmetry will remove the degeneracy of the Γ_8 state resulting a maximum splitting of $^2F_{5/2}$ state into three Kramers doublets. The Kramers doublets are given by $\Gamma_7^{\pm} = \sin\theta|\frac{5}{2}, \pm\frac{5}{2}\rangle + \cos\theta|\frac{5}{2}, \mp\frac{3}{2}\rangle$, where $\sin^2\theta \sim 1/6$, $\Gamma_{8,1}^{\pm} = |\frac{5}{2}, \pm\frac{1}{2}\rangle$, and $\Gamma_{8,2}^{\pm} = \alpha|\frac{5}{2}, \pm\frac{3}{2}\rangle + \sqrt{1-\alpha^2}|\frac{5}{2}, \mp\frac{5}{2}\rangle$, respectively. The CF Hamiltonian for a perfect O_h symmetry is written as $\hat{\mathcal{H}}_{\text{CEF}} = B_4^0\hat{O}_4^0 + B_4^4\hat{O}_4^4 + B_6^0\hat{O}_6^0 + B_6^4\hat{O}_6^4$ where B_n^m are the second, fourth, and sixth order terms and $\hat{O}_m^n$ are the corresponding Stevens operator equivalents[6]. Further constraints includes, $B_4^4 = 5 * B_4^0$ and $B_6^4 = -21 * B_6^4$.In the $J_{\text{eff}} = 1/2$ limit, the maximum allowed terms in the CF Hamiltonian are less than $2J$ meaning that the sixth order terms is zero; B_n^6 = 0, evaluated in the total angular momentum $|j, m_j\rangle$ basis. In this limit, the essential physics is limited to $^2F_{5/2}$ as is the case for Ce^{3+} systems.

CF splitting of Pr^{4+} in $\zeta_{SOC} << \Delta_{CF}$ regime.

The O_h crystal field splits the seven f orbitals to ground state a_{2u}, and excited triply degenerate t_{1u} and t_{2u} states. In the presence of spin-orbit coupling, the seven f orbitals mix yielding 14 KD in line with the 14 states extracted from $|j, m_j\rangle$ states. In the $|m_l, m_s\rangle$ basis, the nature of the Γ_7 KD is given as $|\Gamma_7^{\pm}\rangle = A\sqrt{\frac{6}{7}}|\mp 3, \pm\frac{1}{2}\rangle - B\sqrt{\frac{1}{7}}|\mp 2, \mp\frac{1}{2}\rangle + C\sqrt{\frac{2}{7}}|\pm 1, \pm\frac{1}{2}\rangle - D\sqrt{\frac{5}{7}}|\pm 2, \mp\frac{1}{2}\rangle$, where $\alpha = \frac{A^2+B^2}{C^2+D^2} \sim 0.18$ and $(A^2/B^2)^{\Gamma_7} \sim 2.6$ and $(C^2/D^2)^{\Gamma_7} \sim 0.33$. The first two components of Γ_7 KD ($m_l = -3, -2$) identifies with being derived from $|\frac{5}{2}, \pm\frac{5}{2}\rangle, |\frac{7}{2}, \pm\frac{5}{2}\rangle$, states in $|j, m_j\rangle$ basis, while the last components ($m_l = 1, 2$) identifies with being derived from $|\frac{5}{2}, \pm\frac{3}{2}\rangle, |\frac{7}{2}, \pm\frac{3}{2}\rangle$ states. In the $J_{\text{eff}} = 1/2$ limit, the contributions from $|\frac{7}{2}, \pm\frac{5}{2}\rangle, |\frac{7}{2}, \pm\frac{5}{2}\rangle$ states are negligible with the essential physics limited to $^2F_{5/2}$ SOC manifold as described above. In the $|m_l, m_s\rangle$ basis framework the O_h CF Hamiltonian must be diagonalized with the entire set of 14 LS basis states and thereby making higher-order terms as non-zero, $B_n^6 \neq 0$, while the constraints for B_4^4 and B_6^4 still apply in the O_h symmetry. This framework overcomes the point-charge model established in the $|j, m_j\rangle$ basis and gives a better approximation of the ground state for covalent lanthanide systems. The parameter α defines the ratio of $|j, \pm\frac{5}{2}\rangle$ to $|j, \pm\frac{3}{2}\rangle$. For the original Γ_7 KD, $\alpha \sim 0.25$ indicates the ground state wavefunction is primarily defined by the $m_l = 1, 2$ ($|j, \pm\frac{3}{2}\rangle$) components which agrees well with the Γ_7 wavefucntion derived in the $|j, m_j\rangle$ basis above.

CF splitting of Pr^{4+} in $\zeta_{SOC} \sim \Delta_{CF}$ regime.

Given the large CF energy scale for Pr^{4+} as described in the main text, CF and SOC interactions now compete with each other. Therefore, CF cannot be considered as a perturbation on the SOC energy scale and thereby making the $J_{eff} = 1/2$ picture invalid. The presence of competing interactions drastically changes the single-ion picture which further affects the macroscopic properties of the system as described in the main text. In order to understand the implications of intermediate coupling, we study the evolution of the single-ion properties of Pr^{4+} in the toy model Hamiltonian $\hat{\mathcal{H}}_{\text{CEF}}^{Pr} = B_4^0\hat{O}_4^0 + B_4^4\hat{O}_4^4 + B_6^0\hat{O}_6^0 + B_6^4\hat{O}_6^4$ constrained by O_h symmetry for an isolated PrO_6 unit. We calculate, the eigen energies, ground state wavefucntion composition, α, and g_{av} as a function of B_4^0 for fixed values of B_6^0 as shown in figs S9,S10, and S11. Here, $B_4^0 > 0$ as is expected for a six coordinate system. $B_4^0 < 0$ flips the first excited state quartet as the ground state and the Γ_7 KD as the

excited state and is expected for a eight coordinate system with cubic symmetry as is the case for PrO_2. With B_6^0 = 0 (Fig. S9), for small values of B_4^0, the system can be considered as a traditional lanthanide where the $J_{\text{eff}} = 1/2$ limit still applies. As expected the value of α remains close to 0.25 with the eigen states split as expected for the O_h CF. As B_4^0 increases, the system begins to deviate from the $J_{\text{eff}} = 1/2$ limit and moves towards the $S_{\text{eff}} = 1/2$ where the $\Delta_{CF} >> \zeta_{SOC}$ as described in the main text. For nonphysically large values of B_4^0, the eigen states relax to 3 states where the ground state KD corresponds a_{2u}, the first excited state with three degenerate KD corresponds to triply degenerate t_{1u} and the second excited state with three degenerate KD corresponds to triply degenerate t_{2u}. The values of α tends towards 1 as we move towards the $S_{\text{eff}} = 1/2$ limit. This indicates that, with increase in CF energy scale, the $|j, \pm\frac{5}{2}\rangle$ character adds on to the ground state Γ_7 KD with the final wavefunction in the $S_{\text{eff}} = 1/2$ limit having equal contribution from $|j, \pm\frac{5}{2}\rangle$ and $|j, \pm\frac{3}{2}\rangle$ states. This is also evident from the evolution of g_{av} which shows a dip to almost zero and increases again. As described in the main text, Pr^{4+} systems do exhibit unusually small g values which is in line with intermediate coupling scheme established here.

CF Hamiltonian for Pr^{4+} in 1-Pr.

We begin constraining the Hamiltonian for 1-Pr under a perfect O_h CF while the real symmetry is C_{2h} in order to reduce the number of parameters for fitting, $\hat{\mathcal{H}}_{\text{CEF}}^{1-Pr} = B_4^0\hat{O}_4^0 + B_4^4\hat{O}_4^4 + B_6^0\hat{O}_6^0 + B_6^4\hat{O}_6^4$, where B_4^4, and B_6^4 were constrained under O_h symmetry. Also, B_4^0 was constrained to be > 0 as described in the above section for a six coordinate system. The energy scale of the uncommonly large Δ_{CEF} in 1-Pr is comparable in magnitude to ζ_{SOC} of Pr^{4+}. In the $J_{\text{eff}} = 1/2$ limit, treating the Pr^{4+} ion as isoelectronic Ce^{3+}, $\hat{\mathcal{H}}_{CEF}^{1-Pr}$ was fit to the observed three transitions in INS data. However, we find that these calculations fail to accurately describe the experimental thermo-magnetic data often overestimating. Therefore, we move to intermediate coupling where we diagonalize the $\hat{\mathcal{H}}_{\text{CEF}}^{1-Pr}$ with the entire set of $14LS$ basis states as described above. Initial guesses for the steven's coefficients B_4^0 and B_6^0 were obtained in the $|j, mj\rangle$ basis by setting the first excited state to $E_1^{1-\text{Pr}} = 168$ meV. We note here that, point change based estimation of Steven's coefficients is not appropriate for Pr^{4+} given the anomalously large Pr-$4f$/O-$2p$ covalency. With the initial guesses for B_4^0 and B_6^0, we start fitting the susceptibility data ($T > 50$ K, to avoid the region with short-range correlations) and eigen energies and degeneracies to the Hamiltonian $\hat{\mathcal{H}}_{\text{CEF}}^{1-Pr}$. With the newly estimated values for B_4^0 and B_6^0, we begin to relieve the cubic constraints on B_4^4 and B_6^4 to account for the slight distortion from perfect O_h symmetry. Again, fitting to the susceptibility and eigen energies and degeneracies yields newly estimated values for the stevens coefficients. However, to account for the full distortion from the O_h symmetry, we introduce B_2^0 parameter resulting in a total of 5 independent variables to be fit with the final Hamiltonian being $\hat{\mathcal{H}}_{\text{CF}}^{1-Pr} = B_2^0\hat{O}_2^0 + B_4^0\hat{O}_4^0 + B_4^4\hat{O}_4^4 + B_6^0\hat{O}_6^0 + B_6^4\hat{O}_6^4$. Although, the true symmetry of 1-Pr (C_{2h}) requires $|m| = 2, 6$ in addition to $|m| = 0, 4$ (in B_n^m coefficients), any mixing induced by these parameters would not induce any further loss of degeneracy and hence their effects can be parameterized with $|m| = 0, 4$ parameters. Therefore, we use the truncated Hamiltonian $\hat{\mathcal{H}}_{\text{CF}}^{1-Pr}$. The final fitting was carried out by providing different weights to susceptibility and eigen energies. The final fit parameters and results are provided in Table S3. This yields a set of new KD's with the ground state wavefucntion expressed as a "renormalized" Γ_7 with $\alpha^{1-Pr} = 0.36$. The ground state wavefucniton is given as $|\Gamma_7^{\pm}\rangle = 0.428 \mid \mp 3, \pm\frac{1}{2}\rangle - \; - 0.293 \mid \mp 2, \mp\frac{1}{2}\rangle \; + \; - 0.344 \mid \pm 1, \pm\frac{1}{2}\rangle - \; 0.783 \mid \pm 2, \mp\frac{1}{2}\rangle$.This yields a slightly easy-plane anisotropic g with $g_{xy}^{1-Pr} = 1.37$ and $g_z^{1-Pr} = 0.79$.

CF Hamiltonian for Pr^{4+} in 0-Pr.

We then constrained the Hamiltonian for 0-Pr using a similar method established for 1-Pr. The Hamiltonian was written as $\hat{\mathcal{H}}_{\text{CF}}^{0-Pr} = B_2^0\hat{O}_2^0 + B_4^0\hat{O}_4^0 + B_4^4\hat{O}_4^4 + B_6^0\hat{O}_6^0 + B_6^4\hat{O}_6^4$. Again, the removing the cubic constraints and introduction of B_2^0 parameters is essential to account for the distortion from perfect O_h symmetry. The

final fit parameters and results are provided in Table S3. This yields a set of new KD's with the ground state wavefucntion expressed as a "renormalized" Γ_7 with $\alpha^{0-Pr} = 0.22$. The ground state wavefucniton is given as $|\Gamma_7^{\pm}\rangle = -0.278\,|\mp 3, \pm\frac{1}{2}\rangle - \; 0.314\,|\mp 2, \mp\frac{1}{2}\rangle \; + \; 0.391\,|\pm 1, \pm\frac{1}{2}\rangle - \; -0.819\,|\pm 2, \mp\frac{1}{2}\rangle$. This yields a slightly easy-axis anisotropic g with $g_{xy}^{1-Pr} = 0.63$ and $g_z^{1-Pr} = 1.1$ and is comparable to g values extracted from first principles calculations($g_{xy} \approx 0.7$, $g_z \approx 1.3$).

CF Hamiltonian for Pr^{4+} in 2-Pr.

We then constrained the Hamiltonian for 2-Pr using a similar method established for 1-Pr. The Hamiltonian was written as $\hat{\mathcal{H}}_{\text{CF}}^{0-Pr} = B_2^0\hat{O}_2^0 + B_4^0\hat{O}_4^0 + B_4^4\hat{O}_4^4 + B_6^0\hat{O}_6^0 + B_6^4\hat{O}_6^4$. Again, the removing the cubic constraints and introduction of B_2^0 parameters is essential to account for the distortion from perfect O_h symmetry. The final fit parameters (fitting to susceptibility for $T > 50$ K) and results are provided in Table S3. This yields a set of new KD's with the ground state wavefucntion expressed as a "renormalized" Γ_7 with $\alpha^{2-Pr} = 0.38$. The ground state wavefucniton is given as $|\Gamma_7^{\pm}\rangle = -0.407\,|\mp 3, \pm\frac{1}{2}\rangle - \; 0.331\,|\mp 2, \mp\frac{1}{2}\rangle \; + \; 0.351\,|\pm 1, \pm\frac{1}{2}\rangle - \; - 0.776\,|\pm 2, \mp\frac{1}{2}\rangle$. This yields a slightly easy-axis anisotropic g with $g_{xy}^{1-Pr} = 1.25$ and $g_z^{1-Pr} = 0.37$ and is comparable to g values extracted from first principles calculations($g_{xy} \approx 1.4$, $g_z \approx 0.4$).

XMCD sum rule analysis.

The quantitative sum rule analysis proposed for XAS and XMCD measurement relates the expectation values of the spin ($\langle S_z\rangle$), orbital $\langle L_z\rangle$), and dipole term ($\langle T_z\rangle$) in the valence state. The expression developed by Thole and Carra are given as[7, 8, 9, 10]:

$$\frac{\langle L_z\rangle}{3n_h} = -\frac{\int_{M_4+M_5}\Delta\mu(E)dE}{\frac{3}{2}\int_{M_4+M_5}\mu(E)dE} \tag{S1}$$

$$\langle S_{eff}\rangle = -\frac{3}{2}n_h\frac{\int_{M_5}\Delta\mu(E)dE - \frac{3}{2}\int_{M_4}\Delta\mu(E)dE}{\frac{3}{2}\int_{M_4+M_5}\mu(E)dE} \tag{S2}$$

where, $\langle S_{eff}\rangle = \langle S_z\rangle + 3\langle T_z\rangle$, $\mu(E)$ is the energy dependence of the isotropic XAS, $\Delta\mu(E)$ is the energy dependence of the XMCD, and n_h is the number of holes in the system.Thus once can estimate the orbital and spin moments as $\mu_{orbital} = -\langle L_z\rangle\,\mu_B$, $\mu_{spin} = -2\,\langle S_z\rangle\,\mu_B$, and $\mu_{total} = \mu_{orbital} + \mu_{spin}$. Evaluation of $\langle L_z\rangle$ is straightforward yielding values reported in the main text. However, evaluation of $\langle S_z\rangle$ requires quantitative information about $\langle T_z\rangle$, In most cases, the dipole term is negligible, however for lanthanides with unquenched orbital angular momentum, the dipole term is significant. Therefore, we use the macroscopic bulk magnetization measured at $\mu_{oH} = 5$ T and $T = 20$ K to extract the absolute total moment (μ_{total}). From these values, we can extract μ_{spin} based on the relation $\mu_{total} = \mu_{orbital} + \mu_{spin}$. With this, we can estimate the magnetic dipole contribution based on $\langle S_{eff}\rangle = \langle S_z\rangle + 3\langle T_z\rangle$ with out any sophisticated theoretical modeling.

CF modeling of Pr^{4+} with hybridization.

In order to include hybridization in our CF model, we introduce an orbital reduction parameter κ [11]. The modified CF Hamiltonian is written as $\hat{\mathcal{H}}_{\text{CF}}^{hyb} = \kappa^2\,B_2^0\hat{O}_2^0 + \kappa^4\,B_4^0\hat{O}_4^0 + \kappa^4\,B_4^4\hat{O}_4^4 + \kappa^6\,B_6^0\hat{O}_6^0 + \kappa^6\,B_6^4\hat{O}_6^4$. In this framework, the SOC constant (ζ_{SOC}) is also reduced by κ. Here $\kappa = 1$ corresponds to the mode described in sections 3.1-3.6. By including κ we correct for the metal-ligand covalency driven delocalized states. We begin the fitting process similar to the above method by starting from perfect O_h CF Hamiltonian. We find

initial guesses for B_4^0, B_6^0, and κ until the first excited state is roughly closer to the observed eigen energy. With this, we find a reasonable $\kappa = 0.9$ that accounts for the hybridization. After the initial guess, we fit to the susceptibility data, eigen energies. and eigen degeneracies by allowing all parameters to vary similar to the above model. The value of ζ_{SOC} is always constrained to be κ times the SOC value from the above model. The results obtained from this approach are listed in Table. S8 and the fits are shown in Fig. S12.

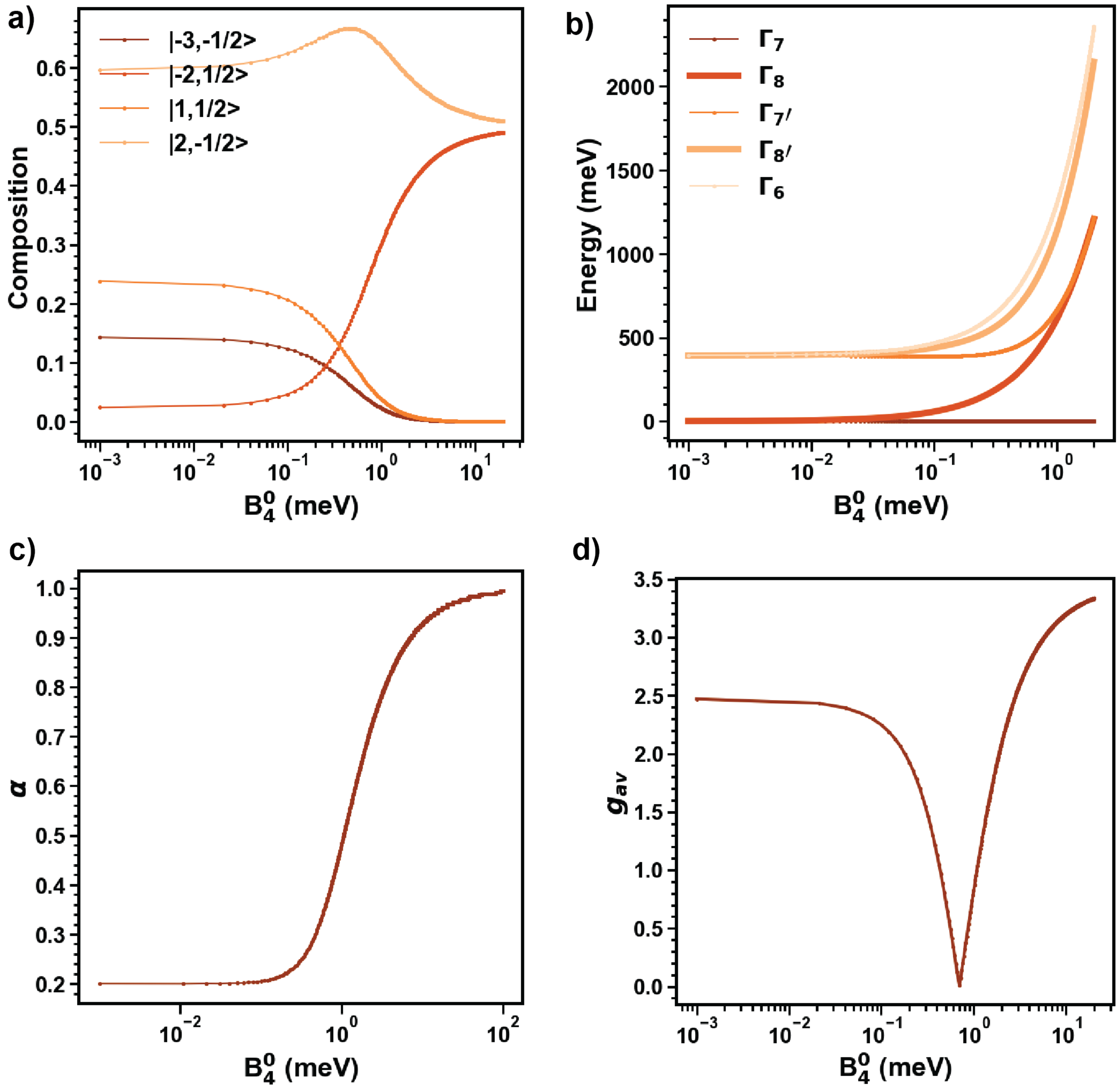

Supplementary Figure 9. Evolution of single-ion properties as a function of B_4^0 with $B_6^0 = 0$. a, Compsition of the ground state wavefucntion. **b**, Eigen energies of the different eigen states relative to the ground state set to 0 meV. The thickness of the lines correspond to the degeneracy of the eigen states. **c**, Evolution of α. **d**, Evolution of g_{av}.

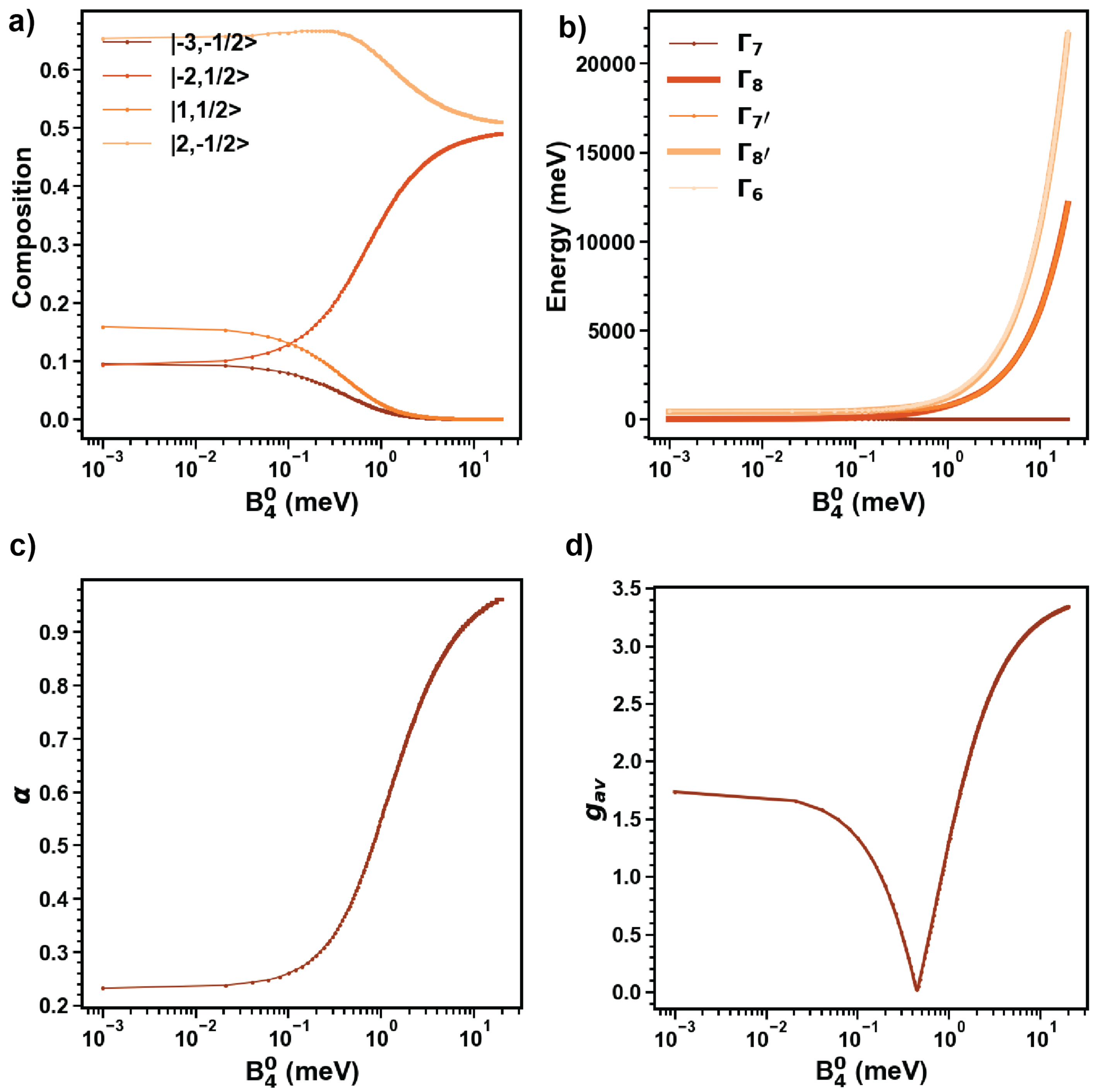

Supplementary Figure 10. Evolution of single-ion properties as a function of B_4^0 with $B_6^0 = 0.01$. a, Compsition of the ground state wavefucntion. **b**, Eigen energies of the different eigen states relative to the ground state set to 0 meV. The thickness of the lines correspond to the degeneracy of the eigen states. **c**, Evolution of α. **d**, Evolution of g_{av}.

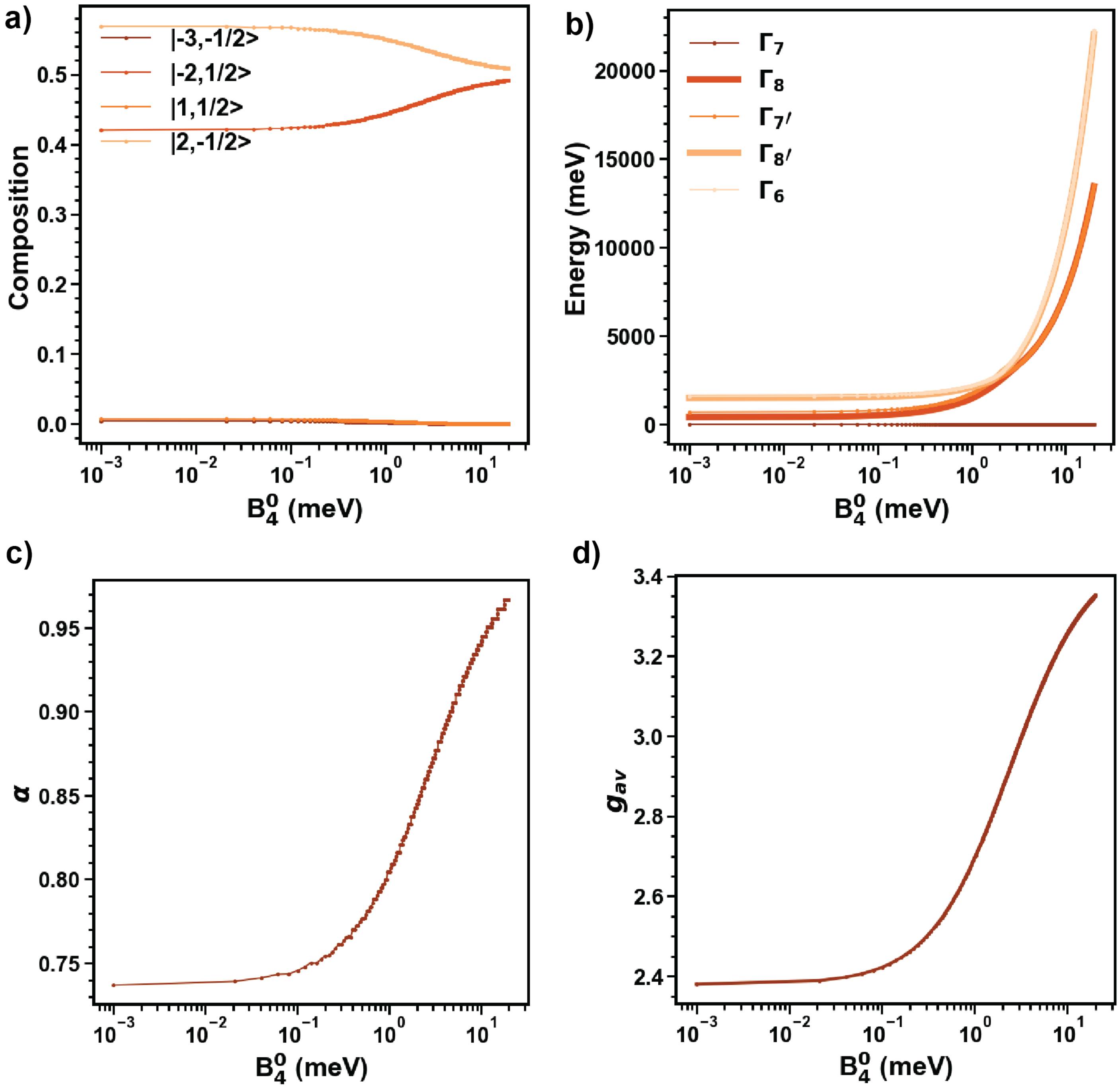

Supplementary Figure 11. Evolution of single-ion properties as a function of B_4^0 with $B_6^0 = 0.1$. a, Compsition of the ground state wavefucntion. **b**, Eigen energies of the different eigen states relative to the ground state set to 0 meV. The thickness of the lines correspond to the degeneracy of the eigen states. **c**, Evolution of α. **d**, Evolution of g_{av}.

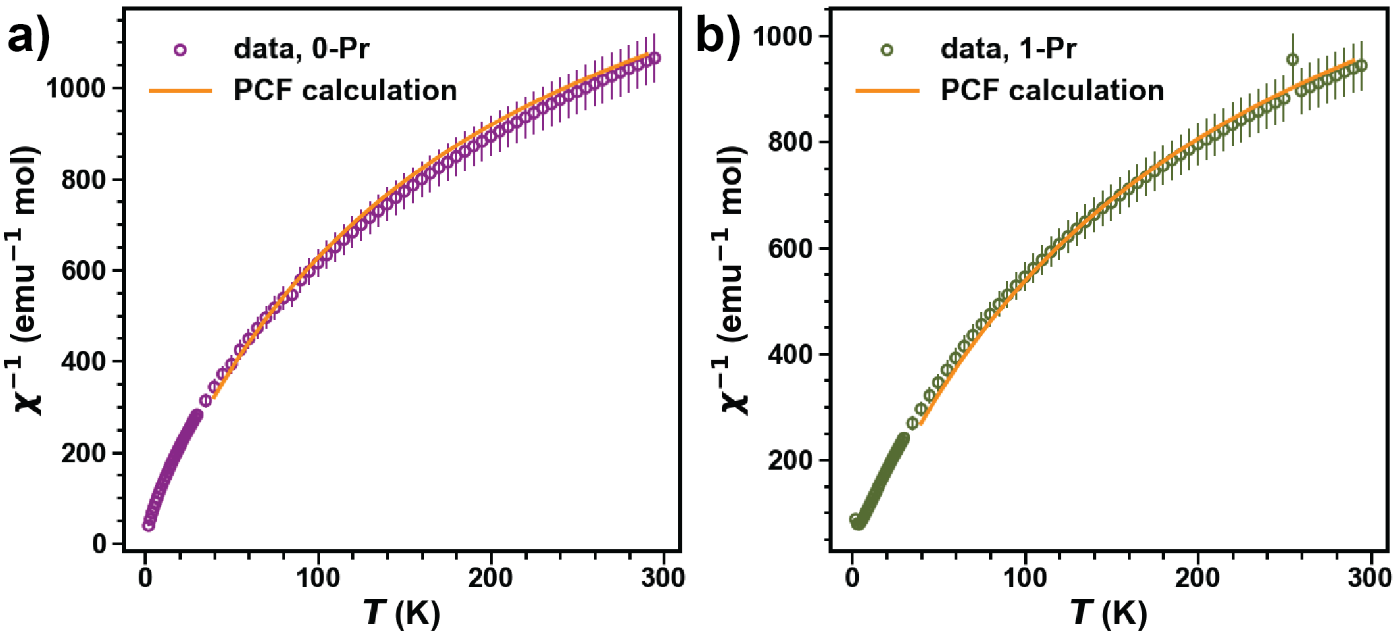

Supplementary Figure 12. CF model with hybridization. a, Susceptibility of 0-Pr and the corresponding calculation. **b**, Susceptibility of 1-Pr and the corresponding calculation.

	Γ_7	0-Pr	1-Pr	2-Pr
B_2^0	*	$1.3(3)$	-12.43	-5.26
B_4^0	*	$0.54(1)$	0.76	0.38
B_4^4	*	$2.29(1)$	3.43	1.85
B_6^0	*	$-0.007(1)$	-0.001	0.003
B_6^4	*	$0.11(1)$	0.45	0.112
ζ_{SOC}	*	112	112	112
g_{av}	≈ 1.4	≈ 0.9	≈ 1.1	≈ 1.1
A[a]	0.352	0.241	0.428	0.407
B[a]	0.215	0.331	0.293	0.331
C[a]	0.454	0.363	0.344	0.351
D[a]	0.79	0.837	0.783	0.776
α[b]	0.18	0.22	0.36	0.37
A^2/B^2	2.6	0.53	2.13	1.51
C^2/D^2	0.33	0.18	0.19	0.20

[a] Coefficients for the ground state wavefucntions as defined in Fig. 1 caption of the main text
[b] α as defined above

Supplementary Table 3. Fit parameters for the three different materials. The small value of B_2^0 for 0-Pr is indicative of how close the PrO_6 unit is close to a perfect O_h symmetry. The true symmetry being S_6 where only a mirror symmetry is broken from the ideal O_h symmetry. Furthermore, the relatively large value of B_6^0 for 0-Pr is indicative of the first excited state being almost 1.5 larger ($\approx$ 266 meV) than for 1-Pr and 2-Pr ($\approx$ 168 meV). All units are in meV.

	0-Pr		1-Pr		2-Pr	
	E^m	E^c	E^m	E^c	E^m	E^c
$KD1$	0	0	0	0	0	0
$KD2$	267	255.1	168	160.8	$*$	155.1
$KD3$	270	260.01	335	328.6	233	244.1
$KD4$	$*$	428.2	387	383.6	$*$	398.1
$KD5$	662	668.8	$*$	832.6	$*$	562.1
$KD6$	$*$	701.3	$*$	923	$*$	640.1
$KD7$	$*$	823.6	$*$	1100	$*$	738.1

[m] Observed from INS or IRMS
[c] PCF Calculation

Supplementary Table 4. Observed and calculated Eigen energies of the different materials studied. All units are in meV.

Supplementary Table 5. Eigenvectors and Eigenvalues for **0-Pr** in $|m_l, m_s\rangle$

E (meV)	$\|-3,-\frac{1}{2}\rangle$	$\|-3,\frac{1}{2}\rangle$	$\|-2,-\frac{1}{2}\rangle$	$\|-2,\frac{1}{2}\rangle$	$\|-1,-\frac{1}{2}\rangle$	$\|-1,\frac{1}{2}\rangle$	$\|0,-\frac{1}{2}\rangle$	$\|0,\frac{1}{2}\rangle$	$\|1,-\frac{1}{2}\rangle$	$\|1,\frac{1}{2}\rangle$	$\|2,-\frac{1}{2}\rangle$	$\|2,\frac{1}{2}\rangle$	$\|3,-\frac{1}{2}\rangle$	$\|3,\frac{1}{2}\rangle$
0.000	0.0	0.241	-0.331	0.0	0.0	0.0	0.0	0.0	0.0	-0.363	0.837	0.0	0.0	0.0
0.000	0.0	0.0	0.0	-0.837	0.363	0.0	0.0	0.0	0.0	0.0	0.0	0.331	-0.241	0.0
255.180	0.0	0.0	0.0	0.0	0.0	0.87	-0.429	0.0	0.0	0.0	0.0	0.0	0.0	-0.243
255.180	0.243	0.0	0.0	0.0	0.0	0.0	0.0	0.429	-0.87	0.0	0.0	0.0	0.0	0.0
260.010	0.0	0.665	-0.6	0.0	0.0	0.0	0.0	0.0	0.0	-0.037	-0.444	0.0	0.0	0.0
260.010	0.0	0.0	0.0	-0.444	-0.037	0.0	0.0	0.0	0.0	0.0	0.0	-0.6	0.665	0.0
428.220	0.0	0.0	0.0	-0.122	-0.562	0.0	0.0	0.0	0.0	0.0	0.0	0.66	0.483	0.0
428.220	0.0	-0.483	-0.66	0.0	0.0	0.0	0.0	0.0	0.0	0.562	0.122	0.0	0.0	0.0
668.860	0.0	0.0	0.0	-0.295	-0.742	0.0	0.0	0.0	0.0	0.0	0.0	-0.309	-0.517	0.0
668.860	0.0	-0.517	-0.309	0.0	0.0	0.0	0.0	0.0	0.0	-0.742	-0.295	0.0	0.0	0.0
701.310	-0.671	0.0	0.0	0.0	0.0	0.0	0.0	0.722	0.17	0.0	0.0	0.0	0.0	0.0
701.310	0.0	0.0	0.0	0.0	0.0	0.17	0.722	0.0	0.0	0.0	0.0	0.0	0.0	-0.671
823.600	0.701	0.0	0.0	0.0	0.0	0.0	0.0	0.542	0.463	0.0	0.0	0.0	0.0	0.0
823.600	0.0	0.0	0.0	0.0	0.0	-0.463	-0.542	0.0	0.0	0.0	0.0	0.0	0.0	-0.701

Supplementary Table 6. Eigenvectors and Eigenvalues for **1-Pr** in $|m_l, m_s\rangle$

E (meV)	$\|-3,-\frac{1}{2}\rangle$	$\|-3,\frac{1}{2}\rangle$	$\|-2,-\frac{1}{2}\rangle$	$\|-2,\frac{1}{2}\rangle$	$\|-1,-\frac{1}{2}\rangle$	$\|-1,\frac{1}{2}\rangle$	$\|0,-\frac{1}{2}\rangle$	$\|0,\frac{1}{2}\rangle$	$\|1,-\frac{1}{2}\rangle$	$\|1,\frac{1}{2}\rangle$	$\|2,-\frac{1}{2}\rangle$	$\|2,\frac{1}{2}\rangle$	$\|3,-\frac{1}{2}\rangle$	$\|3,\frac{1}{2}\rangle$
0.000	0.0	0.41	-0.257	0.0	0.0	0.0	0.0	0.0	0.0	-0.35	0.802	0.0	0.0	0.0
0.000	0.0	0.0	0.0	-0.802	0.35	0.0	0.0	0.0	0.0	0.0	0.0	0.257	-0.41	0.0
160.800	0.0	-0.655	0.488	0.0	0.0	0.0	0.0	0.0	0.0	0.151	0.557	0.0	0.0	0.0
160.800	0.0	0.0	0.0	-0.557	-0.151	0.0	0.0	0.0	0.0	0.0	0.0	-0.488	0.655	0.0
328.690	-0.628	0.0	0.0	0.0	0.0	0.0	0.0	-0.227	0.744	0.0	0.0	0.0	0.0	0.0
328.690	0.0	0.0	0.0	0.0	0.0	0.744	-0.227	0.0	0.0	0.0	0.0	0.0	0.0	-0.628
383.680	0.0	-0.456	-0.827	0.0	0.0	0.0	0.0	0.0	0.0	0.312	0.104	0.0	0.0	0.0
383.680	0.0	0.0	0.0	-0.104	-0.312	0.0	0.0	0.0	0.0	0.0	0.0	0.827	0.456	0.0
832.670	-0.697	0.0	0.0	0.0	0.0	0.0	0.0	0.589	-0.409	0.0	0.0	0.0	0.0	0.0
832.670	0.0	0.0	0.0	0.0	0.0	-0.409	0.589	0.0	0.0	0.0	0.0	0.0	0.0	-0.697
923.240	0.0	0.0	0.0	-0.189	-0.87	0.0	0.0	0.0	0.0	0.0	0.0	-0.108	-0.442	0.0
923.240	0.0	0.442	0.108	0.0	0.0	0.0	0.0	0.0	0.0	0.87	0.189	0.0	0.0	0.0
1100.120	0.0	0.0	0.0	0.0	0.0	0.528	0.776	0.0	0.0	0.0	0.0	0.0	0.0	0.346
1100.120	0.346	0.0	0.0	0.0	0.0	0.0	0.0	0.776	0.528	0.0	0.0	0.0	0.0	0.0

Supplementary Table 7. Eigenvectors and Eigenvalues for **2-Pr** in $|m_l, m_s\rangle$

E (meV)	$\|-3,-\frac{1}{2}\rangle$	$\|-3,\frac{1}{2}\rangle$	$\|-2,-\frac{1}{2}\rangle$	$\|-2,\frac{1}{2}\rangle$	$\|-1,-\frac{1}{2}\rangle$	$\|-1,\frac{1}{2}\rangle$	$\|0,-\frac{1}{2}\rangle$	$\|0,\frac{1}{2}\rangle$	$\|1,-\frac{1}{2}\rangle$	$\|1,\frac{1}{2}\rangle$	$\|2,-\frac{1}{2}\rangle$	$\|2,\frac{1}{2}\rangle$	$\|3,-\frac{1}{2}\rangle$	$\|3,\frac{1}{2}\rangle$
0.000	0.0	-0.418	0.325	0.0	0.0	0.0	0.0	0.0	0.0	0.363	-0.767	0.0	0.0	0.0
0.000	0.0	0.0	0.0	0.767	-0.363	0.0	0.0	0.0	0.0	0.0	0.0	-0.325	0.418	0.0
155.190	0.0	0.749	-0.357	0.0	0.0	0.0	0.0	0.0	0.0	0.004	-0.557	0.0	0.0	0.0
155.190	0.0	0.0	0.0	0.557	-0.004	0.0	0.0	0.0	0.0	0.0	0.0	0.357	-0.749	0.0
244.090	0.338	0.0	0.0	0.0	0.0	0.0	0.0	0.475	-0.813	0.0	0.0	0.0	0.0	0.0
244.090	0.0	0.0	0.0	0.0	0.0	0.813	-0.475	0.0	0.0	0.0	0.0	0.0	0.0	-0.338
398.380	0.0	0.0	0.0	-0.025	-0.365	0.0	0.0	0.0	0.0	0.0	0.0	0.846	0.387	0.0
398.380	0.0	-0.387	-0.846	0.0	0.0	0.0	0.0	0.0	0.0	0.365	0.025	0.0	0.0	0.0
562.960	0.0	0.0	0.0	0.0	0.0	0.039	-0.534	0.0	0.0	0.0	0.0	0.0	0.0	0.845
562.960	-0.845	0.0	0.0	0.0	0.0	0.0	0.0	0.534	-0.039	0.0	0.0	0.0	0.0	0.0
640.850	0.0	0.338	0.224	0.0	0.0	0.0	0.0	0.0	0.0	0.857	0.317	0.0	0.0	0.0
640.850	0.0	0.0	0.0	-0.317	-0.857	0.0	0.0	0.0	0.0	0.0	0.0	-0.224	-0.338	0.0
738.740	0.0	0.0	0.0	0.0	0.0	-0.581	-0.7	0.0	0.0	0.0	0.0	0.0	0.0	-0.415
738.740	-0.415	0.0	0.0	0.0	0.0	0.0	0.0	-0.7	-0.581	0.0	0.0	0.0	0.0	0.0

	Γ_7	0-Pr	1-Pr	2-Pr
B_2^0	*	1.04	-17.11	-7.94
B_4^0	*	0.45	1.12	0.54
B_4^4	*	2.81	5.31	2.13
B_6^0	*	-0.001	-0.008	-0.018
B_6^4	*	0.13	0.80	0.20
ζ_{SOC}	*	100	100	100
g_{av}	≈ 1.4	≈ 0.9	≈ 1.1	≈ 1.1
A[a]	0.352	0.336	0.408	0.413
B[a]	0.215	0.315	0.263	0.298
C[a]	0.454	0.409	0.338	0.37
D[a]	0.79	0.788	0.806	0.777

[a] Coefficients for the ground state wavefucntions as defined in Fig. 1 caption of the main text
[b] α as defined above

Supplementary Table 8. Fit parameters for the three different materials obtained by including hybridization. The small value of B_2^0 for 0-Pr is indicative of how close the PrO_6 unit is close to a perfect O_h symmetry. The true symmetry being S_6 where only a mirror symmetry is broken from the ideal O_h symmetry. Furthermore, the relatively large value of B_6^0 for 0-Pr is indicative of the first excited state being almost 1.5 larger ($\approx$ 266 meV) than for 1-Pr and 2-Pr ($\approx$ 168 meV). All units are in meV.

Supplementary Note 2: First-principles calculations.

In a first step, sets of scalar-relativistic (SR) multiconfigurational wavefunctions were calculated with the complete active space (CAS) self-consistent field approach[12, 13, 14]. Subsequently, these wavefunctions were employed in single-state, multireference CAS second-order perturbation theory (PT2)[15, 16] calculations in order to obtain more accurate energies including effects from dynamic correlation. An imaginary shift of 0.20 au was used with PT2 in order to minimize intruder-state effects. SR effects were introduced with the second-order Douglas-Kroll-Hess Hamiltonian.[17, 18, 19, 20] In a second step, spin-orbit (SO) coupling was introduced using the restricted active-space state-interaction (RASSI) formalism[21] and the atomic mean-field integrals (AMFI).[22] Henceforth, PT2-SR and PT2-SO labels will be used to identify results obtained with only a SR treatment ('spin-free' states belonging to a well-defined spin multiplicity) or with both SR and SO coupling treatments.

Embedding was achieved using a similar strategy as in our previous work on actinide and lanthanide systems.[23, 24] Geometries for the systems under investigation are shown in Fig S13. Within **0-Pr**, the $[PrO_6]^{8-}$ ion is isolated by Li^+ cations and adopts a nearly octahedral geometry (O_h) with a Pr–O bond length of 2.32 Å and bond angles within 90±2.5°. In **2-Pr**, edge-shared $[Pr_2O_10]^{12-}$ dimers occur, in a honeycomb lattice, with structures obeying C_2 symmetry. The individual $[PrO_6]^{12-}$ monomers, labeled with a and b in center panel of Fig S13, both comply with C_2 local symmetry and exhibit geometries that are significantly different between themselves and significantly distorted from O_h. For instance, the Pr–O bond lengths are more than 0.1 Å larger in monomer a vs. b, and the bond angles vary around 90±10° in both monomers.

Supplementary Table 9. Electronic structure of the Pr^{4+} ion.

System	PT2-SR	ΔE	PT2-SO	ΔE
Pr^{4+}	^2F	0.000	$^2F_{5/2}$	0.000
			$^2F_{7/2}$	395

Calculated data for the low-energy electronic states of $[PrO_6]^{12-}$, with PT2-SR and PT2-SO, are collected in Table S10 (isolated O_h structure), Table S11 (structure from 0-Pr) and Table S12 (structures from 2-Pr). Selected natural orbital (NO), natural spin-magnetization orbital (NSO) and spin magnetization plots [$m_W^S(\mathbf{r})$ for quantization along the $W = X$, Y and Z magnetic axes] are shown in Fig S15. Details regarding the generation of NSOs and $m_W^S(\mathbf{r})$ can be found in Reference [25]. Note that the NSO spin populations add up to twice the spin expectation value for a given quantization axis, $2\langle S_W\rangle$, while $m_W^S(\mathbf{r})$ corresponds to the usual spin density if it is calculated without SO coupling. Regardless of the $[PrO_6]^{12-}$ structure considered, the spin-free GS is $^2A_{2u}$ (or of $^2A_{2u}$ parentage for the non-O_h cases) with a single configurational wavefunction derived from the 4f a_{2u}^1 configuration.

Data obtained for the binuclear $[Pr_2O_{10}]^{12-}$ embedded-cluster model of 2-Pr are gathered in Table S13 (energies and magnetism), Fig S16 (localized CAS NOs showing the magnetic orbitals), and in Fig S17 (PT2-SO NOs and populations, NSOs and spin magnetization plots). The lowest energy $S = 1$ spin-triplet and $S = 0$ spin-singlet states are generated by the spin pairing of the 4f a_{2u}^1 electrons localized at the two Pr^{4+} centers (Fig S16). The resulting wavefunctions are single-configurational and described by the Pr_1 $a_{2u}^{\uparrow}$ + Pr_2 $a_{2u}^{\uparrow}$ ($S = 1$) and Pr_1 $a_{2u}^{\uparrow}$ + Pr_2 $a_{2u}^{\downarrow}$ ($S = 0$) configurations. The energy difference obtained with PT2-SR, which identifies with the Heisenberg exchange coupling (J), is 4.2 meV. With SO coupling, there are four low-energy states split by 1.5 meV. These states can be regarded to originate from the GS Kramers doublets

of the individual mononuclear systems. Analysis of the SO wavefunctions showed that the lowest energy SO state has the largest (one-state) contribution (37%) from the lowest energy $S = 0$ spin-free state while the next three SO states with energies ranging between 0.7–1.5 meV have largest (one-state) contributions (also 37%) from the lowest-energy $S = 1$ spin-free state. The strong SO coupling in the GS of the complex is reflected in the NO and NSO populations shown in Fig S17, and also in the small spin expectation value ($\langle S_y \rangle = 0.536$) along the Pr–Pr internuclear axis (magnetic Y-axis, the magnetic axes are also depicted in Fig S17).

Supplementary Table 10. Electronic structure of an O_h $[PrO_6]^{8-}$ isolated free ion.

Atomic multiplet	Spin-free states	ΔE^b	ΔE^c	J multiplet	Kramers doublets SF comp.	ΔE^b	ΔE^c
^2F	$^2A_{2u}$	0.000	0.000	$^2F_{5/2}$	58$^2A_{2u}$ + 42$^2T_{2u}$	0.000	0.000
	$^2T_{2u}$	115	170		54$^2T_{2u}$ + 44$^2T_{1u}$	135	143
	$^2T_{1u}$	252	256		54$^2T_{2u}$ + 44$^2T_{1u}$	135	143
				$^2F_{7/2}$	58$^2T_{2u}$ + 42$^2A_{2u}$	385	397
					53$^2T_{1u}$ + 46$^2T_{2u}$	503	511
					50$^2T_{2u}$ + 46$^2T_{1u}$	503	511
					100$^2T_{2u}$	576	558
Main values of the g tensor in the ground Kramers doublet							
g_X						1.116	0.906
g_Y						1.116	0.906
g_Z						1.116	0.906

[a]The O_h geometry was obtained by symmetrizing the PrO_6 unit of Li_8PrO_6. All energies are in meV units.
[b]State-averaged calculations without symmetry constraint.
[c]State-averaged calculations with D_{2h} symmetry constraint.

Supplementary Table 11. Electronic structure of a $[PrO_6]^{8-}$ unit of **0-Pr**.[a]

Atomic multiplet	Spin-free states	ΔE^b	ΔE^c	J multiplet	Kramers doublets SF comp.^{c}	ΔE^b	ΔE^c
^2F	$^2A_{2u}$	0.000	0.000	$^2F_{5/2}$	65^2A_{2u} + 35^2T_{2u}	0.000	0.000
	$^2T_{2u}$	129	158		76^2T_{2u} + 24^2T_{1u}	141	241
		129	184		80^2T_{2u} + 20^2T_{1u}	151	246
		148	184				
	$^2T_{1u}$	270	498	$^2F_{7/2}$	65^2T_{2u} + 35^2A_{2u}	388	396
		272	544		65^2T_{1u} + 20^2T_{2u}	513	662
		273	544		80^2T_{1u} + 20^2T_{2u}	516	707
					100^2T_{1u}	588	820
Main values of the g tensor in the ground Kramers doublet							
g_X						1.015	0.714
g_Y						1.018	0.714
g_Z						1.095	1.229

[a] All energies are in eV units. Selected orbital isosurfaces and populations are shown in Figure 15.
[b] $[PrO_6]^{8-}$ isolated ion of Fig S13 (top panel).
[c] $[PrO_6]^{8-}$ embedded-cluster model of Fig S13 (top panel).
[d] Magnetic axes are depicted in Fig S15.

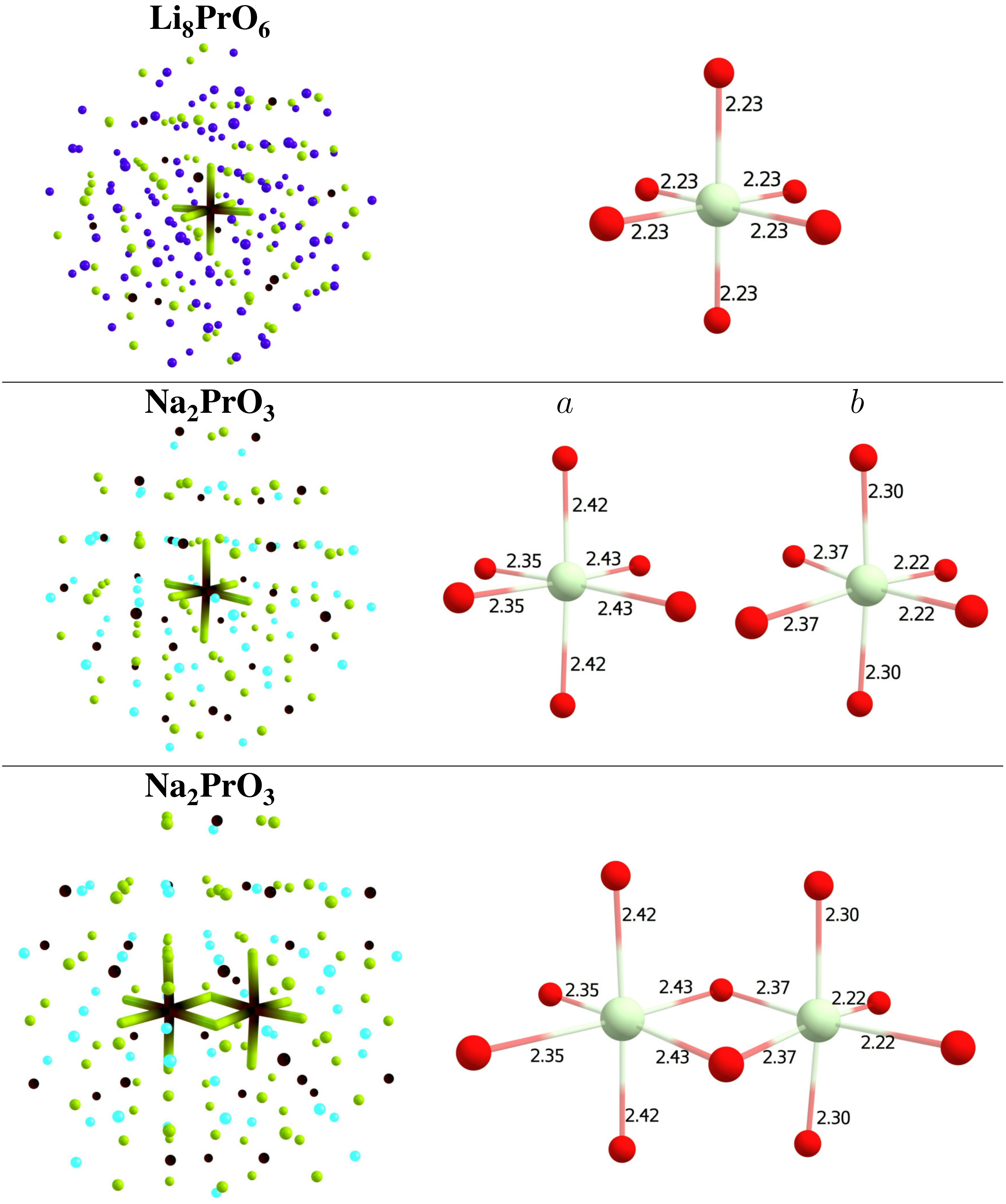

Supplementary Figure 13. Embedded cluster models and bare ion geometries used in the present study: $[PrO_6]^{8-}$ and $[Pr_2O_{10}]^{12-}$ with surrounding embedding pseudocharges (left panels) and without (right panels).

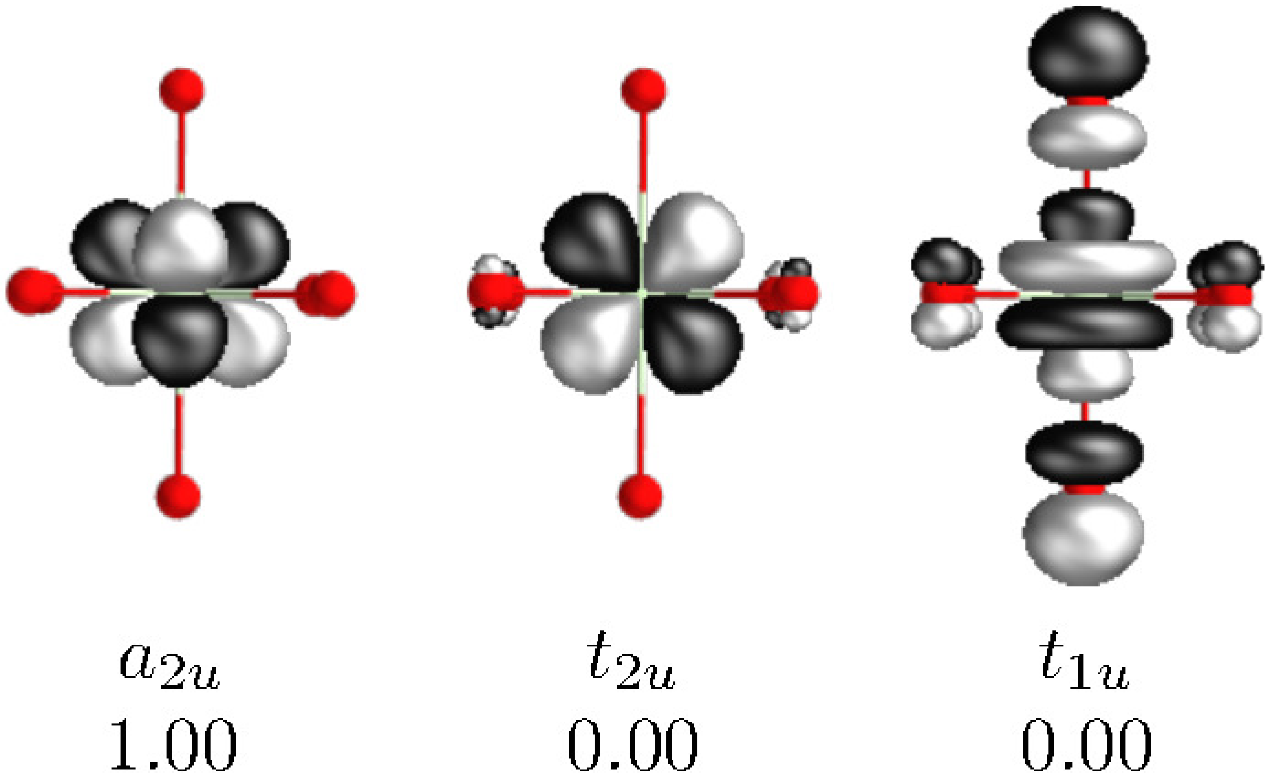

Supplementary Figure 14. Ground-state natural orbitals: Ground-state natural orbitals (±0.03 au iso-surfaces) of Pr $4f$ AO parentage and corresponding populations calculated with CAS-SR for an O_h $[PrO_6]^{8-}$ isolated ion with a Pr–O bond length of 2.23 Å.).

Supplementary Table 12. Electronic structure of the $[\mathrm{PrO}_6]^{8-}$ embedded crystal-model system of **2-Pr**.[a]

Atomic multiplet	Spin-free states				J multiplet	Kramers doublets			
		ΔE^b	ΔE^c	ΔE^d		SF comp.[d]	ΔE^b	ΔE^c	ΔE^d
^2F	$^2A_{2u}$	0.000	0.000	0.000	$^2F_{5/2}$	$57^2A_{2u} + 43^2T_{2u}$	0.000	0.000	0.000
						$63^2T_{2u} + 37^2T_{1u}$	118	136	168
	$^2T_{2u}$	089	118	096		$69^2T_{2u} + 31^2T_{1u}$	162	163	231
		099	120	099					
		156	159	201	$^2F_{7/2}$	$59^2T_{2u} + 38^2A_{2u}$	387	389	394
						$63^2T_{1u} + 37^2T_{2u}$	479	498	536
	$^2T_{1u}$	214	248	323		$77^2T_{1u} + 23^2T_{2u}$	529	537	623
		237	268	349		$94^2T_{1u} + 6^2T_{2u}$	597	601	754
		306	312	502					
Main values of the g tensor in the ground Kramers doublet[d]									
g_X							1.694	1.490	1.772
g_Y							1.649	1.310	1.536
g_Z							0.110	0.406	0.049
Expectation values[d]									
Magnetic X-axis direction[e]									
$\langle L_x \rangle$							−1.359	−1.265	−1.429
$\langle S_x \rangle$							0.256	0.260	0.268
Magnetic Y-axis direction[e]									
$\langle L_y \rangle$							−1.312	−1.155	−1.315
$\langle S_y \rangle$							0.243	0.250	0.254
Magnetic Z-axis direction[e]									
$\langle L_z \rangle$							−0.306	−0.558	0.217
$\langle S_z \rangle$							0.125	0.177	−0.119

[a] All energies are in eV units. Selected orbital isosurfaces and populations are shown in Figure S15.
[b] Structure a in Fig S13.
[c] Structure b in Fig S13.
[d] Structure a plus embedding in Fig S13.
[e] Magnetic axes are depicted in Fig S15.

Supplementary Table 13. Electronic structure of the $[Pr_2O_{10}]^{12-}$ embedded crystal-model system of **2-Pr**.

Atomic multiplet	Spin-free states	ΔE (eV)	J multiplet	Spin-orbit states	ΔE (eV)
$^2F^{\mathrm{Pr}_1} + {}^2F^{\mathrm{Pr}_2}$	$S = 0$	0.0000	$^2F^{\mathrm{Pr}_1}_{5/2} + {}^2F^{\mathrm{Pr}_2}_{5/2}$	37% ($S = 0$)	0.0000
	$S = 1$	0.0042		37% ($S = 1$)	0.0007
				37% ($S = 1$)	0.0008
				37% ($S = 1$)	0.0015
Heisenberg exchange coupling (J)		4.2 meV			≈1 meV[a]
Expectation values					
Magnetic X-axis direction[b]					
$\langle L_x \rangle$					−2.767
$\langle S_x \rangle$					0.566
Magnetic Y-axis direction[b]					
$\langle L_y \rangle$					−2.444
$\langle S_y \rangle$					0.536
Magnetic Z-axis direction[b]					
$\langle L_z \rangle$					−0.296
$\langle L_x \rangle$					0.367
$\langle S_z \rangle$					0.090

[a]With SO coupling, spin is not a good quantum number.
[b]Magnetic axes are depicted in Figure S17. The magnetic Y-axis corresponds to the Pr–Pr internuclear axis.

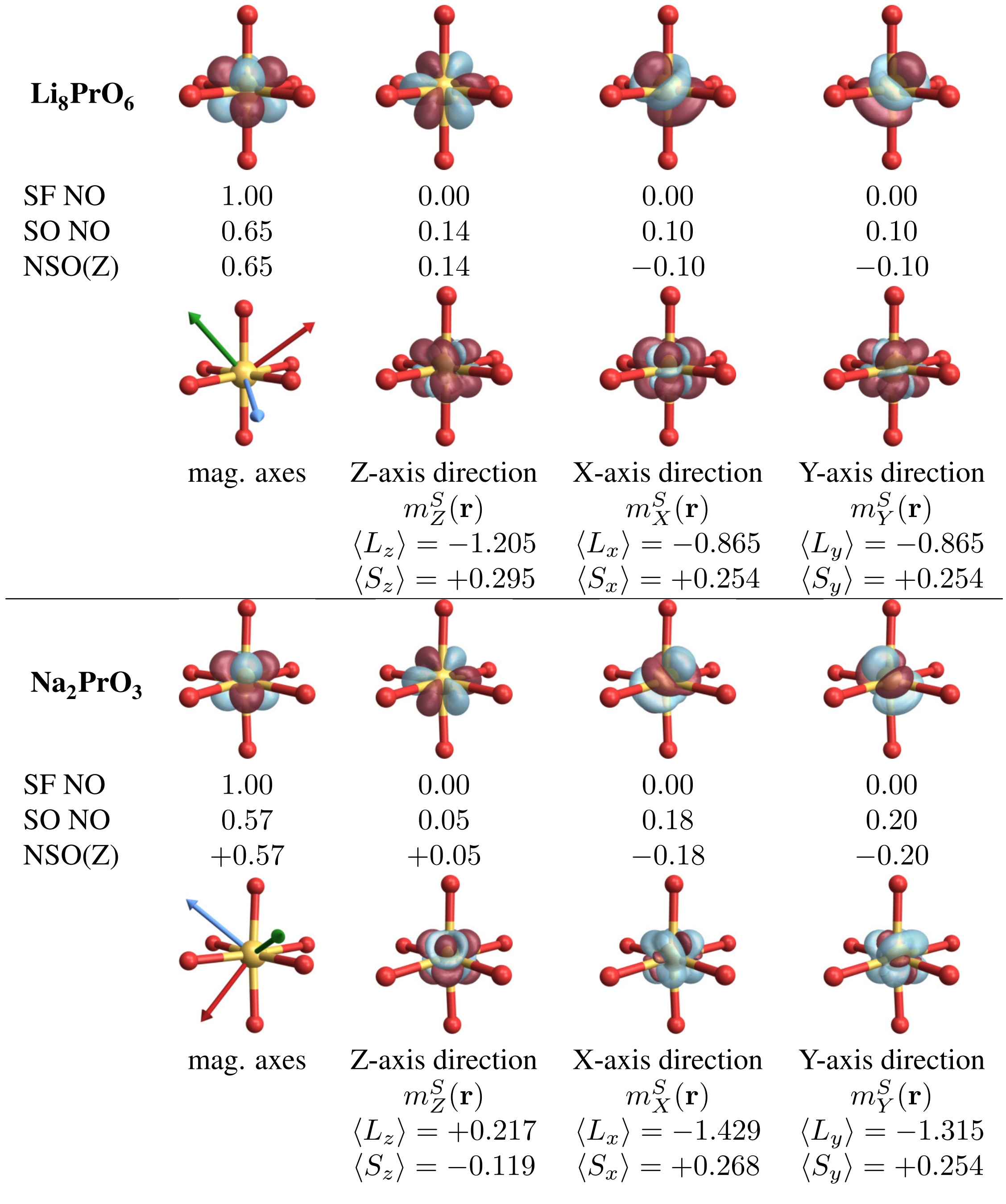

Supplementary Figure 15. Selected natural orbitals (NOs, ±0.03 au isosurfaces) and their populations, with and without SO coupling, calculated for the $[PrO_6]^{8-}$ embedded cluster model of **0-Pr** (top panel) and **2-Pr** (bottom panel). Also given are the spin populations corresponding to the natural spin orbitals (NSOs, isosurfaces identical with those of the NOs) calculated for the Z direction of the spin magnetization, along the magnetic Z-axis, for the GS Kramers component with $\langle S_z \rangle > 0$. Plots of the spin magnetization $[m^S_W(\mathbf{r})]$ for quantization along the $W = X$, Y and Z magnetic axes are also given with isosurface of ±0.001 au. Color code for magnetic axes: red for Z-axis, green for X-axis, blue for Y-axis.

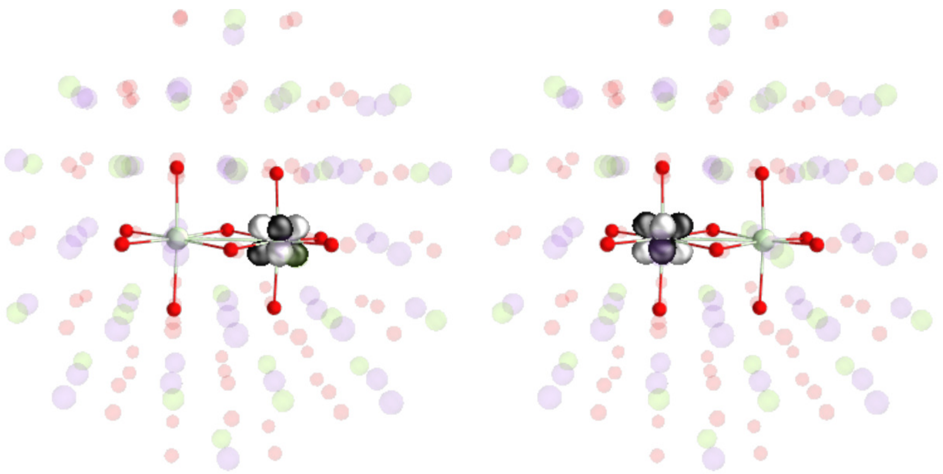

$S = 1$ ↑ ↑ weight = 100%

$S = 0$ ↑ ↓ weight = 100%

Supplementary Figure 16. Localized CAS orbitals characterizing the electron pairing in the lowest-energy spin-triplet ($S = 1$) and spin-singlet ($S = 0$) configurations of $[Pr_2O_{10}]^{12-}$ embedded-cluster model of Na_2PrO_3. The localization was achieved by arbitrary rotations among the CAS NOs and a subsequent CAS configuration interaction was performed to tailor the wavefunction configurational admixture.

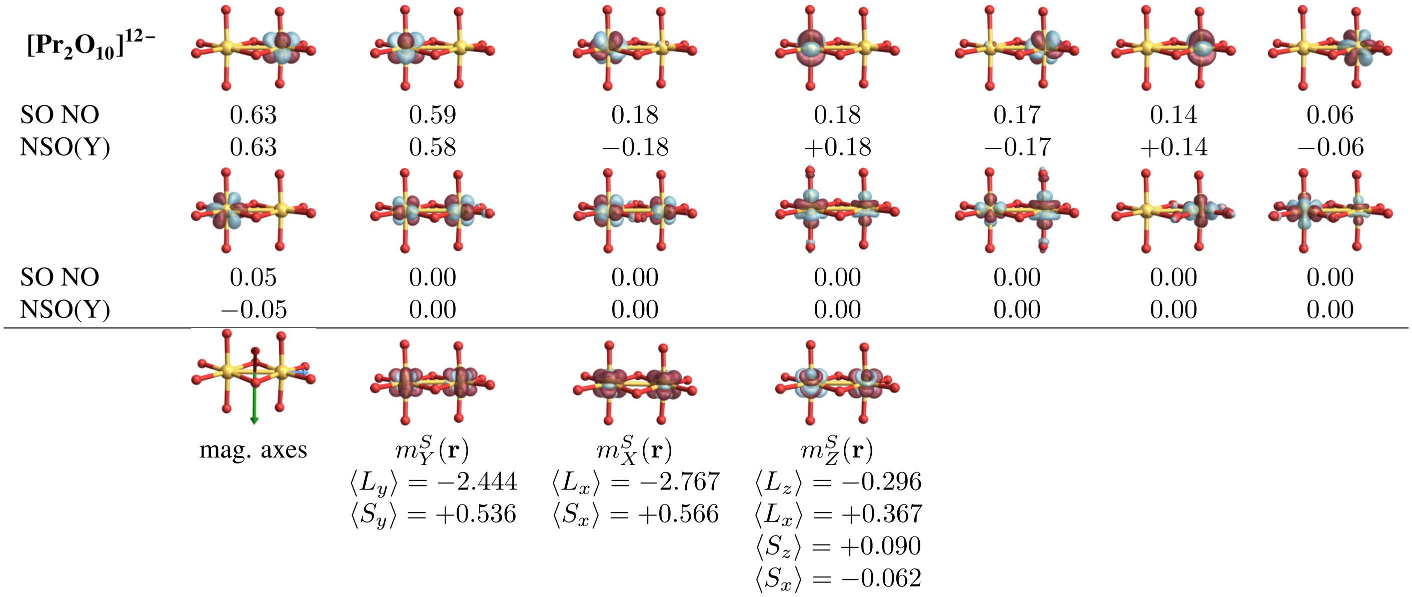

Supplementary Figure 17. Top panel: Selected natural orbitals (NOs, ± 0.03 au isosurfaces) and their populations calculated with PT2-SO for the $[Pr_2O_{10}]^{12-}$ embedded cluster model of **2-Pr**. Also given are the spin populations corresponding to the natural spin orbitals (NSOs, isosurfaces identical with those of the NOs) calculated for the Y direction of the spin magnetization, along the magnetic Y-axis. Bottom panel: Plots of the spin magnetization $[m_W^S(\mathbf{r})]$ for quantization along the $W = X$, Y and Z magnetic axes are also given with isosurface of ± 0.001 au. Color code for magnetic axes: red for Z-axis, green for X-axis, blue for Y-axis.

Supplementary References

[1] Ramanathan, A., Leisen, J. E. & La Pierre, H. S. In-plane cation ordering and sodium displacements in layered honeycomb oxides with tetravalent lanthanides: $\mathrm{Na_2LnO_3(Ln = Ce,\ Pr,\ and\ Tb)}$. *Inorganic Chemistry* **60**, 1398–1410 (2021).

[2] Coelho, A. A. Topas and topas-academic: an optimization program integrating computer algebra and crystallographic objects written in c++. *Journal of Applied Crystallography* **51**, 210–218 (2018).

[3] Scheie, A. Pycrystalfield: software for calculation, analysis and fitting of crystal electric field hamiltonians. *Journal of Applied Crystallography* **54**, 356–362 (2021).

[4] Minasian, S. G. *et al.* Quantitative evidence for lanthanide-oxygen orbital mixing in $\mathrm{CeO_2}$, $\mathrm{PrO_2}$, and $\mathrm{TbO_2}$. *Journal of the American Chemical Society* **139**, 18052–18064 (2017).

[5] Cowan, R. D. *The theory of atomic structure and spectra.* 3 (Univ of California Press, 1981).

[6] Stevens, K. Matrix elements and operator equivalents connected with the magnetic properties of rare earth ions. *Proceedings of the Physical Society. Section A* **65**, 209 (1952).

[7] Thole, B. *et al.* $3d$ x-ray-absorption lines and the $3d^94f^{n+1}$ multiplets of the lanthanides. *Physical Review B* **32**, 5107 (1985).

[8] Tripathi, S. XMCD investigation at $M_{4,5}$ edges of the rare earth elements in high-performance permanent magnet (2018).

[9] Thole, B., Carra, P., Sette, F. & van der Laan, G. X-ray circular dichroism as a probe of orbital magnetization. *Physical Review Letters* **68**, 1943 (1992).

[10] Carra, P., Thole, B., Altarelli, M. & Wang, X. X-ray circular dichroism and local magnetic fields. *Physical Review Letters* **70**, 694 (1993).

[11] King, D. M. *et al.* Molecular and electronic structure of terminal and alkali metal-capped uranium (v) nitride complexes. *Nature communications* **7**, 13773 (2016).

[12] Roos, B. O., Taylor, P. R. & Siegbahn, P. E. M. A Complete Active Space SCF method (CASSCF) using a density matrix formulated super-CI approach. *Chem. Phys.* **48**, 157–173 (1980).

[13] Olsen, J., Roos, B. O., Jørgensen, P. & Jensen, H. J. A. Determinant based Configuration-Interaction algorithms for complete and restricted Configuration-Interaction spaces. *J. Chem. Phys.* **89**, 2185–2192 (1988).

[14] Malmqvist, P. Å., Rendell, A. & Roos, B. O. The restricted active space self-consistent-field method, implemented with a split graph unitary group approach. *J. Phys. Chem.* **94**, 5477–5482 (1990).

[15] Andersson, K., Malmqvist, P.-Å., Roos, B. O., Sadlev, A. J. & Wolinski, K. Second-order perturbation theory with a CASSCF reference function. *J. Phys. Chem.* **94**, 5483–5488 (1990).

[16] Angeli, C., Cimiraglia, R., Evangelisti, S., Leininger, T. & Malrieu, J.-P. Introduction of n-electron valence states for multireference perturbation theory. *J. Chem. Phys.* **114**, 10252–10264 (2001).

[17] Douglas, M. & Kroll, N. M. Quantum electrodynamical corrections to the fine structure of helium. *Ann. Phys.* **82**, 89–155 (1974).

[18] Hess, B. A. Applicability of the no-pair equation with free-particle projection operators to atomic and molecular structure calculations. *Phys. Rev. A* **32**, 756–763 (1985).

[19] Hess, B. A. Relativistic electronic-structure calculations employing a two-component no-pair formalism with external-field projection operators. *Phys. Rev. A* **33**, 3742–3748 (1986).

[20] Wolf, A., Reiher, M. & Hess, B. A. The generalized Douglas-Kroll transformation. *J. Chem. Phys.* **117**, 9215–9226 (2002).

[21] Malmqvist, P.-A., Roos, B. O. & Schimmelpfennig, B. The restricted active space (ras) state interaction approach with spin-orbit coupling. *Chem. Phys. Lett.* **357**, 230–240 (2002).

[22] Hess, B. A., Marian, C. M., Wahlgren, U. & Gropen, O. A mean–field spin–orbit method applicable to correlated wavefunctions. *Chem. Phys. Lett.* **251**, 365–371 (1996).

[23] Gendron, F. & Autschbach, J. Puzzling lack of temperature dependence of the puo_2 magnetic susceptibility explained according to ab-initio wavefunction calculations. *J. Phys. Chem. Lett.* **8**, 673–678 (2017). URL https://doi.org/10.1021/acs.jpclett.6b02968.

[24] Sergentu, D.-C., Booth, C. H. & Autschbach, J. Probing multiconfigurational states by spectroscopy: The cerium xas L_3-edge puzzle. *Chem. Eur. J.* **27**, 7239–7251 (2021).

[25] Gendron, F. *et al.* Magnetic properties and electronic structure of neptunyl^{vi} complexes: Wavefunctions, orbitals, and crystal-field models. *Chem. Eur. J.* **20**, 7994–8011 (2014).
